# Entrustable professional activities in medical education: strategies and results - a systematic review

**DOI:** 10.1080/10872981.2026.2740871

**Published:** 2026-10-10

**Authors:** Nuno Fernandes, Clarinda Neves, Helena Donato, José Miguel Pêgo, João Carlos Ribeiro

**Affiliations:** a Faculdade de Medicina, University of Coimbra, Coimbra, Portugal; b Internal Medicine Department - Centro Hospitalar Baixo Vouga, Aveiro, Portugal; c Biomedical Sciences Department - University of Aveiro, Aveiro, Portugal; d Unidade Local de Saúde de Coimbra. Serviço de Documentação e Informação Científica, Hospitais da Universidade de Coimbra, Coimbra, Portugal; e Life and Health Sciences Research Institute (ICVS), School of Medicine, University of Minho, Braga, Portugal; f ICVS/3B's, PT Government Associate Laboratory Braga, Guimarães, Portugal

**Keywords:** Entrustable professional activities, systematic review, medical education, competence-based medical education, health care quality, access and evaluation

## Abstract

**Background:**

Medical education has been marked in the last decades by the introduction of Competence-Based Medical Education (CBME). Despite its clear objectives, CBME has faced significant challenges and Entrustable Professional Activities (EPAs) have been conceptualised as an answer to some of those challenges.

**Aims/Purposes:**

This systematic review aimed primarily to summarise the evidence published on the strategies used for the development, implementation, and assessment of EPAs and their results. Secondarily this review also aimed to characterise the body of evidence produced on EPAs, assess its quality, and explore its limitations and future research paths.

**Methods:**

Systematic searches were conducted in six electronic databases in July 2021 and updated in March 2025. The data extracted included the quality assessment of the studies considered using the Quality Assessment Tool for Studies with Diverse Designs (QATSDD).

**Results:**

Four hundred and seventy-eight studies were included for data extraction and posterior analysis, most of which referred to Postgraduate programmes (59.6%, *n* = 285) and took place in the USA (51.5%, *n* = 246). A relevant diversity of methodologies and stakeholders involved was detected in the development, implementation, and assessment of EPAs. References to factors influencing the implementation of EPAs (50.8%, *n* = 243) and the potential of implementing EPAs for the improvement of learning and assessment practices (46.4%, *n* = 222) were found in a significant number of studies, but only a small number focused on the cost-effectiveness of the use of EPAs. The quality analysis of the included studies revealed modest results.

**Conclusions:**

This broad systematic review highlights the increasing adoption of EPAs, showcasing the diversity of contexts and strategies for the development, implementation, and assessment of EPAs. Nevertheless, significant challenges persist, namely concerning study quality, warranting further research.

## Introduction

The evolution of medical education is closely linked to the evolution of medicine and important landmarks can be found through the centuries, laying the foundations for Competence-Based Medical Education (CBME). Early models of medical education were largely unregulated and linked to ambiguous notions of proficiency instead of clearly defined competencies. Over time, however, more explicit expectations regarding performance were progressively introduced as a consequence of the appearance of state regulation, licensing examinations, and formal curricula [1–3].

During the twentieth century, medical education systems faced the need to implement reforms to answer to the expansion of scientific knowledge and increasing societal expectations regarding patient safety, quality of healthcare and professional accountability. During this period curricula became more content-heavy, more time-fixed and discipline-based, aiming at a more comprehensive coverage rather than demonstrable outcomes. These changes led to frequent curricular overload and to the implicit assumption that time spent in training was an adequate indicator of competence.

The focus on competency-based approaches following World War II, made it possible to address the issue of curricular overload and the need for more efficient training, resulting in a reassessment and redesign of curricula [1], by shifting the focus from time-based progression to clear learning outcomes.

The last decades of the 20^th^ century and the first decades of the 21^st^ century witnessed important progress in medical education that favoured the implementation of CBME thanks to numerous contributions, namely the development of operational frameworks such as Milestones and Entrustable Professional Activities (EPAs), supported by improvements in assessment theory and digital systems [1,4,5].

### CBME as the medical education framework of choice

Since George Miller emphasised the need for practical competence, and several authors have called for increased accountability and patient safety focus, CBME has emerged as the framework of choice in medical education [6,7]. CBME focuses on continuous progress through evaluation, self-directed learning and feedback, aligning with adult learning theories [8].

Although CBME is widely accepted, conceptualisation and operationalization issues remain to be solved [4,9]. EPAs aim at addressing these issues, bridging the knowledge gap between theoretical competencies and ‘real-world’ training needs by applying the key concepts of CBME to the workplace context in a pragmatic way with high acceptance of the main stakeholders [10–13].

### Characteristics and history of EPAs

According to O. ten Cate and colleagues, EPAs consist of three key elements: 1) ‘Entrustable,’ which refers to tasks that learners may complete with varying degrees of supervision; 2) ‘Professional,’ which refers to tasks requiring a variety of specialised professional competencies; and 3) ‘Activities,’ which are professional tasks expected of a qualified individual [14].

EPAs aim at operationalizing several competencies in clinical practice by integrating them holistically, rather than replacing them. They serve as fundamental professional activities, requiring learners to demonstrate a range of competencies that are directly witnessed in clinical settings [15], replacing traditional time-based training with a tailored performance-based advancement, and emphasising outcomes rather than the process [12,14].

Initially developed for postgraduate medical education (PGME), EPAs have now expanded into undergraduate medical education (UGME) and the rest of the medical education continuum [16,17].

Despite the growing use of EPAs, several challenges remain present. The lack of conceptual coherence can lead to discrepancies during the development and implementation of EPAs resulting in results difficult to interpret. The difficulties with the assessment validity and involvement of key stakeholders can additionally endanger the validity, reproducibility and acceptability of EPAs causing difficulties for its wide adoption and justifying focused efforts to address them effectively. [15,18,19].

### Justification for our systematic review, objectives and aims

Previous comprehensive analyses have identified important gaps in the understanding of EPAs and its application across various educational settings [20–29]. This review addressed important problems concerning the development, implementation, and evaluation of EPAs.

Therefore, this systematic review aimed to achieve the following:

- Explore the impact assessment and validation of EPAs, and the feasibility of their adoption.
- Identify the most common practices used for the development, implementation, and evaluation of EPAs.
- Examine how technology and human resources are used for the development, implementation and evaluation of EPAs, and how different stakeholders are involved in these processes.
- Analyse the later stages of EPAs adoption and update on the current knowledge on the entrustment procedure.
- Identify the concepts and frameworks that were most explored in previous research, with a special focus on the evolution and implementation of EPAs in various contexts of the medical education continuum.
- Explore the key limitations of the previous studies.
- Offer potential future research directions to optimise the application of EPAs.
- Assess the characteristics and quality of the published research on EPAs.

## Materials and methods

### Design and research team

Systematic reviews are recognised for their ability to consolidate diverse research findings, offering a more objective perspective than other review methodologies do [30–36]. Given those characteristics we conducted this systematic review to provide an up-to-date, reliable resource on EPAs and registered our study on the PROSPERO platform.

The research team of this study was composed of 3 clinicians with extensive dedication to Postgraduate Medical Education (NF, CN, JCR) and 2 clinicians with a long dedication to Undergraduate Medical Education (JMP, JCR), all of whom have different clinical backgrounds and are considered experts in medical education. The research group also integrated 1 medical librarian with a vast expertise in systematic review searching (HD).

We adhered to the Preferred Reporting Items for Systematic Review and Meta-analysis (PRISMA) guidelines [37] both for conducting and reporting this systematic review.

Given the high heterogeneity and diversity of the included studies, no meta-analysis was conducted.

### Search strategy

In July 2021, we conducted initial searches in six electronic databases: PubMed, Embase (via Elsevier), the Cochrane Library Central Register of Controlled Trials (CENTRAL), ERIC (via EBSCO), CINAHL (via EBSCO), and the Web of Science Core Collection. In March 2025, an update to the initial search was conducted to gather the most recent literature. Since most articles from the initial search were found in the PubMed, the referred update only took into consideration articles from this database.

The search strategy focused on terms specific to the scope of this review. Keywords included were ‘entrustable professional activity,’ ‘Entrustable professional activities,’ and ‘EPAs,’ linked with the Boolean operator ‘OR’. These were further linked to the term ‘medical education’ using the Boolean operator ‘AND’ to refine the focus (for additional detail, see Supplement 1).

The search temporal limits were set from 2005 onwards, in line with the introduction of the EPAs concept in medical education.

The search strategy was developed under the coordination of the researcher with most expertise in systematic review search (HD).

### Study selection

#### Inclusion criteria

We focused primarily on original articles that addressed EPAs. The inclusion criteria considered were:

- **Population**: Studies involving various medical education groups, reporting on undergraduate, postgraduate, fellowship, and continuous medical education programmes.
- **Intervention and Focus**: Studies analysing the exposure to EPAs framework.
- **Comparator/Control**: When reported, studies comparing the EPAs framework to traditional educational frameworks.
- **Outcome explored**:
  ∘ Impact of EPAs on educational outcomes.
  ∘ Methodology for the development of EPAs.
  ∘ Methodology for the implementation of EPAs.
  ∘ Methodology for the assessment of EPAs.
  ∘ Quality assessments of the developed EPAs.
- **Study design:** Eligible study designs included randomised controlled trials, noncontrolled intervention studies, pilot studies, pre-post intervention studies, observational studies, and educational case reports.
- **Setting and Language:** Studies analysing EPAs on medical education settings and published in English, French, Spanish and Portuguese.

#### Exclusion criteria

We decided to exclude studies that did not meet the inclusion criteria previously described.

Additional reasons considered for the exclusion of the studies were as follows:

- **Study Design:** Review articles, commentary, or opinion articles.
- **Type of Publication:** Conference proceedings, theses (Master’s or PhD)
- **Accessibility**: Articles lacking available abstracts and/or full texts.

### Screening process

Following the search strategy, two authors (NF and CN) independently screened titles and abstracts according to predefined inclusion and exclusion criteria. Papers not meeting these criteria were excluded, with reasons for exclusion documented systematically. Discrepancies between the two reviewers were addressed through periodic meetings where each case was discussed until a consensus was reached. If a consensus could not be reached, a third author (JCR) was consulted to resolve the disagreement and finalise the decision.

The screening process was accomplished by the two authors using the Rayyan® web tool [38].

### Data extraction

After the screening process, the papers selected were searched for full text availability.

The data extraction phase commenced with a pilot test of 10 randomly chosen papers to develop, refine and pilot the data extraction tool. The data extraction tool was developed using the Microsoft® Excel® software [39] and included categories focused on the characteristics of the studies included, methodologies used for the assessment of the impact of the use of EPAs, the development of EPAs, the implementation of EPAs, the assessment of EPAs and conceptual frameworks explored. The extraction tool also included categories exploring the limitations of the studies included, the research gaps and future research opportunities identified, as well as the quality of the EPAs reported, and the studies included.

NF and CN independently conducted data extraction and coding, achieving a 98% agreement. In cases of disagreement, discussions were held until a consensus was reached. If consensus was not attained, a third author (JCR) was consulted to make the final decision.

### Studies quality assessment

The Quality Assessment Tool for Studies with Diverse Designs (QATSDD) was chosen to assess the quality of the studies in this review, due to its proven effectiveness in numerous reviews on medical education. [29,40].

### Data analysis

For most of the variables, the data analysis was limited to frequency analysis. When considered appropriate, analyses of measures of central tendency and dispersion were also performed. The statistical analysis was accomplished using SPSS® software [41].

### Artificial intelligence tools usage

During the finalisation of the text of this systematic review, artificial intelligence tools were used exclusively for writing improvement and rephrasing. No artificial intelligence tools were used for content generation. The artificial intelligence tool ChatGPT-4® developed by OpenAI [42] was used for writing improvement. The QuillBot® tool [43] was also used for writing improvements and rephrasing.

## Results

### Search, screening and inclusion of studies

Following the identification and screening of potential articles, 478 articles met all the criteria and were included in the final systematic review [5,16,18,19,44–517]. The details of this process can be found in the PRISMA Flow Diagram (Figure 1).

**Figure 1.**
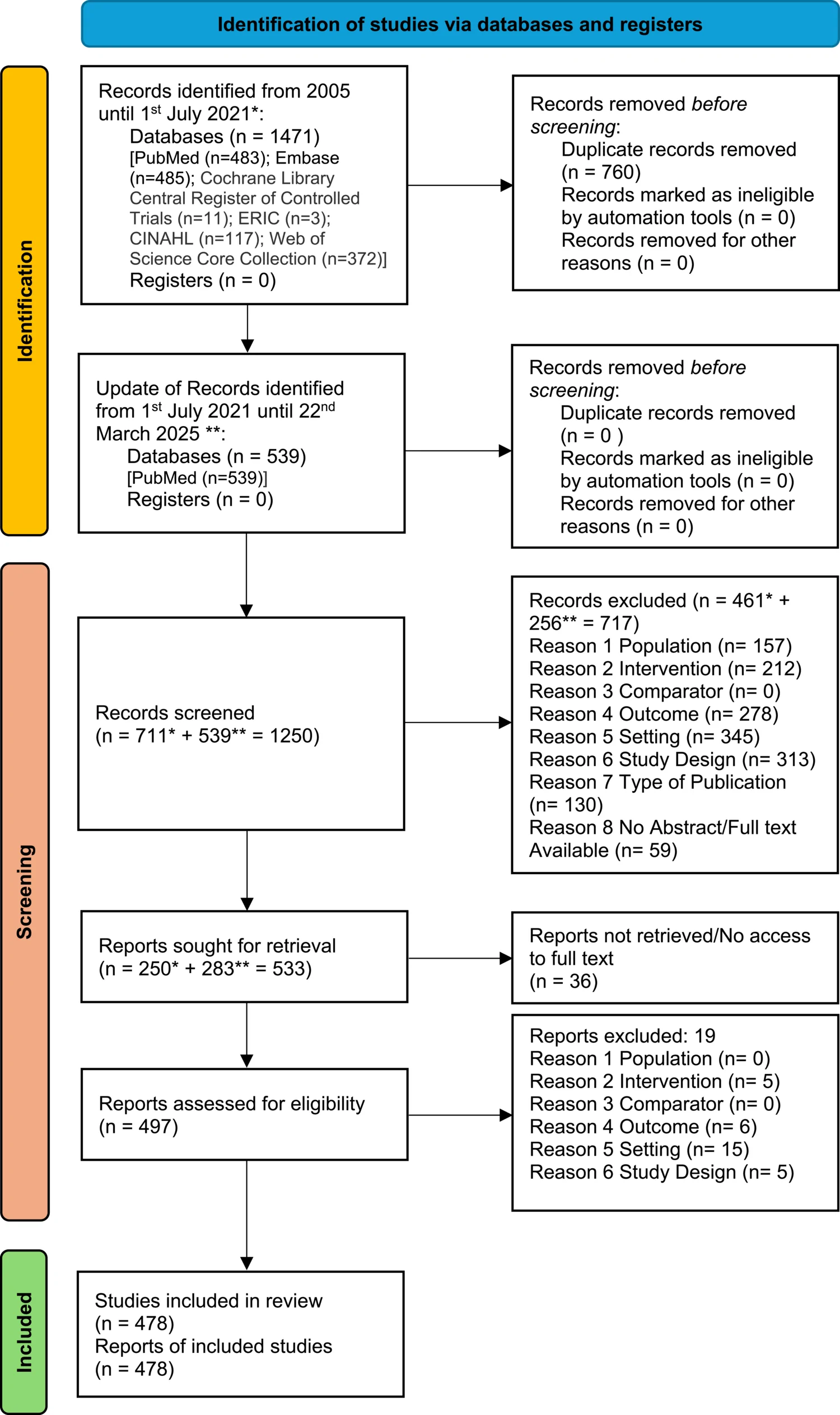
Prisma Flow Diagram. * Records identified from 2005 until 1^st^ July 2021. ** Update of records identified from 1^st^ July 2021 until 22^nd^ March 2025.

### Main characteristics of the studies included

Following the aim of exploring the characteristics of the studies included some of the main characteristics are summarised in Table 1 (for details on individual studies, see Supplement 2).

Most of the studies referred to PGME (*n* = 285; 59.6% of the studies) and took place in the USA (*n* = 246; 51.5%) in single-centre settings (*n* = 256; 53.6% of the studies). Additionally, the percentage of studies involving Asian, African and South American countries was very low.

A total of 46 medical education fields were represented. The General Undergraduate Medical Education (UGME General) and Paediatrics settings accounted for 24.5% (*n* = 117) and 11.5% (*n* = 55) of the studies included respectively.

The studies selected were published from 2011 until 2025, and the percentage of studies has increased over the years.

Observational design accounted for the majority of the studies designs detected and observational cross-sectional design was identified in 34.7% (*n* = 166) of the cases.

With respect to transparency and ethics principles, we found that the funding source was reported in 67.2% (*n* = 321) of the studies, a Declaration of Conflict of interests was presented in 76.6% (*n* = 366) of the studies and, in 74.9% (*n* = 358) of the studies, references were found to the previous analysis and approval by an Ethics committee.

A registration record was detected in 28.4% (*n* = 136) of the cases.

English was the language of publication in 99.2% (*n* = 474) of the studies.

**Table 1.** Main characteristics of the studies included.

	Number of studies	% of studies
**Stages of Medical Education**		
PGME	285	59,6%
UGME	144	30,1%
Fellowship	56	11,7%
Continuous medical education	9	1,9%
Master Programme (Post Graduation Programme)	1	0,2%
No information	3	0,6%
**Country**		
USA	246	51,5%
Canada	93	19,5%
Multiple countries	31	6,5%
Netherlands	27	5,7%
Germany	22	4,6%
Switzerland	16	3,3%
India	11	2,3%
Australia	9	1,9%
China	7	1,5%
Taiwan	6	1,3%
Ireland	5	1,1%
Countries referred in < 1% of the studies*	34	7,1%
**Single-centre/Multicenter Study**		
Single-Centre	256	53,6%
Multicenter	222	46,4%
**Field of medicine focused**		
UGME General	117	24,5%
Paediatrics	55	11,5%
Surgery	48	10%
Internal Medicine	47	9,8%
Multiple Specialities	46	9,6%
Emergency Medicine	37	7,7%
Anaesthesiology	22	4,6%
Psychiatry	14	2,9%
Family medicine	12	2,5%
General surgery	8	1,7%
Orthopaedics	5	1,1%
Nephrology	5	1,1%
Fields of medicine referred in < 1% of the studies**	131	27,4%
No information	1	0,2%
**Year of Publication**		
2011	1	0,2%
2012	1	0,2%
2013	3	0,6%
2014	9	1,9%
2015	10	2,1%
2016	19	4%
2017	25	5,2%
2018	29	6,1%
2019	38	8%
2020	55	11,5%
2021	66	13,8%
2022	46	9,6%
2023	61	12,8%
2024	88	18,4%
2025	27	5,6%
**Study design (Type)**		
Observational Cross-sectional	166	34,7%
Observational Cohort study (Focus Group)	101	21,1%
Observational Cohort study (Consensus)	100	20,9%
Observational Cohort Study (Prospective)	36	7,5%
Observational Cohort Study (Retrospective)	35	7,3%
Observational Case control	1	0,2%
Educational Case report	110	23%
Interventional Clinical Trial (non-RCT)	92	19,2%
Interventional Clinical Trial (RCT)	10	2,1%
Validation study	55	11,5%
Literature review	48	10%
Mixed method	134	28%
Others	1	0,2%
**Study funding report**		
Yes	321	67,2%
No information	157	32,8%
**Study Conflicts of interests**’ **declaration**		
Yes	366	76,6%
No information	112	23,4%
**Study evaluation by Ethics committee declaration**		
Yes	358	74,9%
No information	120	25,1%
**Study registration record**		
Yes	136	28,4%
No information	342	71,6%
**Main Language of publication**		
English	474	99,2%
French	2	0,4%
Spanish	2	0,4%

Abbreviations: PGME, Postgraduate Medical Education; UGME, Undergraduate Medical Education

^*^ Countries referred in <1% of the studies: Ethiopia, Japan, Mexico, Iran, Brazil, Saudi Arabia, Pakistan, UK, New Zealand, Argentina, Sweden, Belgium, South Africa, Singapore, Indonesia, Nepal, France, Hungary, Afghanistan, Colombia.

^**^ Fields of medicine referred in <1% of the studies: Urology, Physical Medicine and Rehabilitation, Primary care, Radiotherapy, Rheumatology, Radiology, Pathology, Critical care, Sports Medicine, Ears Nose and Throat (ENT), Obstetrics and Gynaecology, Gastroenterology, Medical Education, UGME Rotations, Oncology, Palliative Medicine, Geriatrics, PGME Transitional Year, Apheresis Medicine, Cardiology, Infectious Disease, Osteopathic Medicine, Community Medicine, Haematology-Oncology, General practice, Neurosurgery, Neurology, Endocrinology, Dermatology, Vascular Surgery, Ophthalmology, Genetics, Plastic Surgery.

### Journal of publication and quartile analysis of journal of publication

The characteristics of the scientific journals and their quartile analysis can be found in Table 2 (Table 2. Journal of publication and Quartile analysis, presented as Supplement 3).

### Main focus of studies

Continuing the assessment of the main characteristics of the studies, their main focuses are summarised in Table 3. One of the main focuses of 52.9% (*n* = 253) of the studies was the Implementation of EPAs, whereas the Development of EPAs was found to be a main focus of 33.3% (*n* = 159).

**Table 3.** Main focus of studies.

	Number of studies	% of studies
**Main Focus of Studies**		
Implementation of EPAs	253	52,9%
Development of EPAs	159	33,3%
Assessment of EPAs	152	31,8%
Impact assessment of implementation of EPAs	57	11,9%

### Characteristics of the methodologies used to assess the impact of the use of EPAs

Another aim of our review was to analyse the strategies used to assess the impact of the use of EPAs. The results of that analysis are presented in Table 4.

In 51.5% (*n* = 246) of the studies references were found to the assessment of Knowledge, Skills and Attitudes of those exposed to the EPAs framework.

The assessment of the Entrustment or Supervision level was explored in 52.9% (*n* = 253) of the studies included. The tool or scale of entrustment used was detailed in 44.4% (*n* = 212) of the studies included, and the 5 level entrustment scale, as described by Olle ten Cate, was referenced in 14.4% (*n* = 69) of the studies.

Stakeholders’ satisfaction was assessed in 28% (*n* = 134) of the studies, while feasibility was addressed in 29.9% (*n* = 143) and cost-effectiveness of EPAs use in 4.4% (*n* = 21) of studies.

References were made to the improvement of the learning and assessment methodologies as a result of the use of EPAs in 46.4% (*n* = 222) of the studies.

**Table 4.** Methodologies used to assess the impact of EPAs.

	Number of studies	% of studies
**Assessment of Knowledge/Skills/Attitudes**		
Yes	246	51.5%
No information	232	48.5%
**Assessment Entrustment/Supervision level**		
Yes	253	52.9%
No information	225	47.1%
**Tool/Scale of Entrustment Used**		
O Ten Cate 5 level scale	69	14.4%
Other multilevel scale	131	27.4%
Ottawa entrustment scale	7	1.5%
Yes/no scale	8	1.7%
No information	266	55.6%
**Assessment of Satisfaction stakeholders**		
Yes	134	28%
No information	344	72%
**Assessment of Feasibility**		
Yes	143	29.9%
No information	335	70.1%
**Assessment of Cost-effectiveness**		
Yes	21	4.4%
No information	457	95.6%
**Assessment of Improvement of learning/assessment methods**		
Yes	222	46.4%
No information	256	53.6%

### Characteristics of the methodologies used for the development of EPAs

One of the aims of our review was to explore the most common practices used for the development of EPAs and the main results are summarised in Table 5.

The most common methodologies used were consensus methods (*n* = 95; 19.9%) and focus group techniques (*n* = 91; 19% of the studies).

Different stakeholders were identified in the development of EPAs, and Medical education experts and Clinicians were the 2 most referred stakeholders (20.7% (*n* = 99) and 17.2% (*n* = 82) of the studies respectively).

**Table 5.** Characteristics of development of EPAs.

	Number of studies	% of Studies
**Methodology used for the Development of EPAs (Type)**		
Consensus methods	95	19,9%
Focus group	91	19%
Literature review	58	12,1%
Survey	25	5,2%
EPAs mapped to Milestones/Competencies	23	4,8%
EPAs benchmarked with other sets of EPAs	19	4%
Steps O. Ten Cate recommendation	13	2,7%
Structured interviews	9	1,9%
EPAs merged with previous EPAs	2	0,4%
EPAs tested in clinical setting	1	0,2%
Other	4	0,8%
No information	305	63,8%
**Stakeholders involved in the Development of EPAs**		
Medical education experts	99	20,7%
Clinicians	82	17,2%
Residency programmes directors/coordinators	62	13%
Students/residents	59	12,3%
Faculty representatives	49	10,2%
Patients representatives	13	2,7%
Professional association representatives	9	1,9%
Fellows	5	1%
Health administration representatives	4	0,8%
Others	13	2,7%
No information	306	64%

### Characteristics of methodologies used for the implementation of EPAs

Continuing the aim to identify the most common practices used, Table 6 summarises the characteristics of the methodologies used for the implementation of EPAs.

The diverse methodologies considered for the Implementation of EPAs were explored in 60.3% (*n* = 288) of the studies identified. The observation by Faculty members of the execution of EPAs in the workplace was referenced in 42.3% (*n* = 202) of the studies, and the observation of EPAs in Simulated setting was referenced in 19.2% (*n* = 92) of the studies.

In 50.8% (*n* = 243) of the studies, factors such as logistical difficulties (29.5%, *n* = 141) were reported as potentially influencing EPAs implementation.

The strategies used for Stakeholders Development (particularly for Faculty members) were explored in 19.2% (*n* = 92) of the studies, with Presential Workshop/‘Hands-on’ Workshops being reported in 7.1% (*n* = 34) of the studies.

In 26.4% (*n* = 126) of the studies, the use of technology media for the implementation of EPAs was reported.

**Table 6.** Characteristics of Implementation of EPAs.

	Number of studies	% of studies
**Methodologies used for Implementation of EPAs (Type)**		
Observation by faculty in workplace	202	42.3%
Observation in simulated setting	92	19.2%
EPAs performance recorded in Portfolio	34	7.1%
Teaching sessions/Lectures attendance	31	6.5%
Enrolment in CBME curriculum with EPAs	13	2.7%
Records evaluation	28	5.9%
Self-learning (written materials, videos…)	20	4.2%
Scientific divulgation activities	18	3.8%
360 feedback	16	3.3%
Peer feedback	8	1.7%
Research activities	6	1.3%
Teaching/Formation activities	3	0.6%
Others	8	1.7%
No information	190	39.7%
**Factors Influencing Implementation of EPAs (Type)**		
Logistical difficulties	141	29.5%
Faculty development issues	75	15.7%
Stakeholders’ engagement issues (ex: faculty engagement with educational programme)	50	10.5%
EPAs construction issues	50	10.5%
Learning environment	42	8.8%
Stakeholders’ acceptance of educational programme	30	6.3%
Assessment opportunities	28	5.9%
Student/resident versus Faculty relationship issues	24	5%
Students/residents development issues	20	4.2%
Cases complexity	14	2.9%
Students/residents personal characteristics	12	2.5%
Type of evaluation considered (ex: Formative versus Summative)	6	1.3%
Type of evaluator (ex: Faculty versus residents)	5	1%
Collegiality	1	0.2%
Others	11	2.3%
No information	235	49.2%
**Strategies used for Stakeholders/Faculty Development**		
Presential Workshop/‘Hands-on’ Workshop	34	7.1%
Presential Lecture-based Learning session	28	5.9%
Web based learning programme	28	5.9%
Simulation-based training sessions	13	2.7%
Orientation Guide	13	2.7%
Self-oriented literature exploration	11	2.3%
Group work	8	1.7%
Others	3	0.6%
No information	386	80.8%
**Technology Media Used for Implementation of EPAs**		
Website based platform	97	20.3%
Mobile App based platform	37	7.7%
Others	1	0.2%
No information	352	73.6%

### Characteristics of methodologies used for the assessment of EPAs

The identification of the most common methodologies used for the assessment of EPAs was also one of the objectives of our review, and Table 7 summarises the most relevant results.

Practice evaluation by faculty in the workplace was the most common assessment method of EPAs, referred in 44.6% (*n* = 213) of the studies.

Global Entrustment Scales and standardised rubrics were the most used tools for the Assessment of EPAs (referred in 30.8% (*n* = 147) and 20.9% (*n* = 100) of the studies respectively).

There were references to Validation efforts of the Assessment Tools used in 12.8% (*n* = 61) of the studies identified, and the characteristics of the evaluators of the EPAs were explored in 61.7% (*n* = 295) of the studies.

**Table 7.** Assessment of EPAs.

	Number of studies	% of studies
**Methodology used for the Assessment of EPAs (Type)**		
EPA observed in practice by faculty	213	44.6%
Non Clinical/Simulated performance	91	19%
Presentations/Clinical discussions evaluation	42	8.8%
Written examination	39	8.2%
Portfolio review	37	7.7%
Chart based audit	34	7.1%
360 feedback	20	4.2%
Comparison with control group	2	0.4%
Others	5	1%
No information	192	40.2%
**Tools used for the Assessment of EPAs (Type)**		
Global entrustment scale	147	30.8%
Standardised rubric	100	20.9%
Assessment form	32	6.7%
Self-evaluation scale	5	1%
Structured feedback from non-faculty stakeholders	3	0.6%
Number of errors	2	0.4%
No information	244	51%
**Validation of Tools used for the assessment**		
Yes	61	12.8%
No information	417	87.2%
**Characteristics of evaluators of EPAs (Type)**		
Faculty	279	58.4%
Self-evaluation	72	15.1%
Peers (Other students/residents)	47	9.8%
Other health care professionals	26	5.4%
Standardised patients	11	2.3%
Patients	4	0.8%
Others	3	0.6%
No information	183	38.3%
**Characteristics of Assessment (Formative/Summative)**		
Formative	109	22.8%
Summative	88	18.4%
No information	325	68%
**Timming of the assessment**		
Various moments during ME intervention	198	41.4%
End of ME intervention	46	9.6%
Beginning of ME intervention	2	0.4%
No information	233	48.7%

### Phase of development and implementation of EPAs, and conceptual frameworks explored

Our review also aimed to analyse the phases of the development and implementation of EPAs explored in the studies included and, as shown in Table 8, in 60.5% (*n* = 289) of the studies, the focus was on the initial phases of the development and implementation of EPAs.

The identification of the conceptual frameworks related to medical education most explored was another objective of our research, and Table 9 summarises those results.

**Table 8.** Phases of development and implementation of EPAs.

	Number of studies	% of studies
**Phases of development/implementation of EPAs**		
Initial	289	60.5%
Advanced	117	24.5%
No information	72	15%

**Table 9.** Conceptual frameworks explored.

	Number of studies	% of studies
EPAs	478	100%
CBME	295	61.7%
AAMC Core EPAs	130	27.2%
ACGME Core Competencies	79	16.5%
CanMEDs	66	13.8%
Milestones	66	13.8%
ACGME milestones	65	13.6%
Miller’s pyramid	14	2.9%
Core EPAs from scientific professional societies	5	1%
Ottawa Criteria mapping	2	0.4%
Others	2	0.4%

Abbreviations: AAMC, Association of American Medical Colleges; ACGME, Accreditation Council for Graduate Medical Education.

### Limitations of the studies

The exploration of the key limitations of the studies included was also defined as an objective for our review, and Table 10 presents the summary of those limitations. Issues with generalisability of results (52.7%, *n* = 252), issues with study design (49.4%, *n* = 236), and potential biases (42.5%, *n* = 203) were some of the limitations most commonly identified.

**Table 10.** Limitations of the studies.

	Number of studies	% of studies
**Limitations Identified**		
Issues with generalisability of results	252	52.7%
Issues with study design	236	49.4%
Potential biases	203	42.5%
Single/few centres setting	193	40.4%
Small sample size	114	23.8%
Single speciality setting	104	21.8%
Issues with data quality/quantity	101	21.1%
Issues with validity/reliability of results	89	18.6%
Narrow context applicability	84	17.6%
Sampling method	81	17%
Issues with validity/reliability of assessment	81	17%
Issues with participants profile	72	15.1%
Issues related to novelty of CBME and EPAs	43	9%
Issues with methodology	40	8.4%
Low response rate of participants	39	8.2%
Uniqueness of organisational/culture setting	13	2.7%
Participants demographics	11	2.3%
Others	3	0.6%
No information	78	16.3%

### Research gaps and future research opportunities

The identification of research gaps was also defined as a goal of our research with the intention to point out possible future research opportunities. The summary of the results is presented in Table 11.

The need to Evaluate the Impact of EPAs implementation, to Increase the Generalisability of the results and to Explore Different Assessment Strategies were referenced in 43.3% (*n* = 207), 41.2% (*n* = 197) and 26.6% (*n* = 127) of the studies, respectively.

**Table 11.** Research gaps and future research opportunities.

	Number of studies	% of studies
**Research gaps and future opportunities**		
Impact evaluation of EPAs implementation	207	43.3%
Need to increase generalisability	197	41.2%
Exploration of different assessment strategies	127	26.6%
Exploration of stakeholders’ development	102	21.3%
Evaluation of influencing factors of EPAs implementation	101	21.1%
Investigation of validity/reliability	83	17.4%
Real setting evaluation	78	16.3%
Inclusion of different stakeholders	73	15.3%
Definition of best practices	66	13.8%
Need for development and evaluation of data systems	59	12.3%
Evaluation of main challenges and barriers to implementation of EPAs	42	8.8%
Exploration of influencing factors of entrustment decisions	42	8.8%
Analysis/comparison of different interventions/strategies	28	5.9%
Exploration of challenges related to entrustment decisions	11	2.3%
Others	4	0.8%
No information	116	24.3%

### Quality assessment of the reported EPAs

The majority of the studies had no reference to any strategy for the assessment of quality of EPAs (*n* = 439; 92% of the studies) as shown in Table 12.

**Table 12.** Quality assessment of EPAs reported.

	Number of studies	% of studies
**Strategies/Tools used Quality assessment of EPAs**		
EQual	18	3.7%
O. Ten Cate development principles	18	3.7%
QUEPA	3	0.6%
No information	439	92%

Abbreviations: EQual, Queen’s EPA Quality Rubric; QUEPA, Quality of Entrustable Professional Activities.

### Studies quality assessment using QATSDD tool

The summary of the quality assessment of the studies is presented in Table 13 (Table 13. Quality assessment using QATSDD, presented as Supplement 4).

Most of the studies included were Qualitative studies (47.5% of the studies; *n* = 227).

Figure 2. presents the details of the evolution of the percentage of the maximum possible QATSDD score of the studies included over the years.

**Figure 2.**
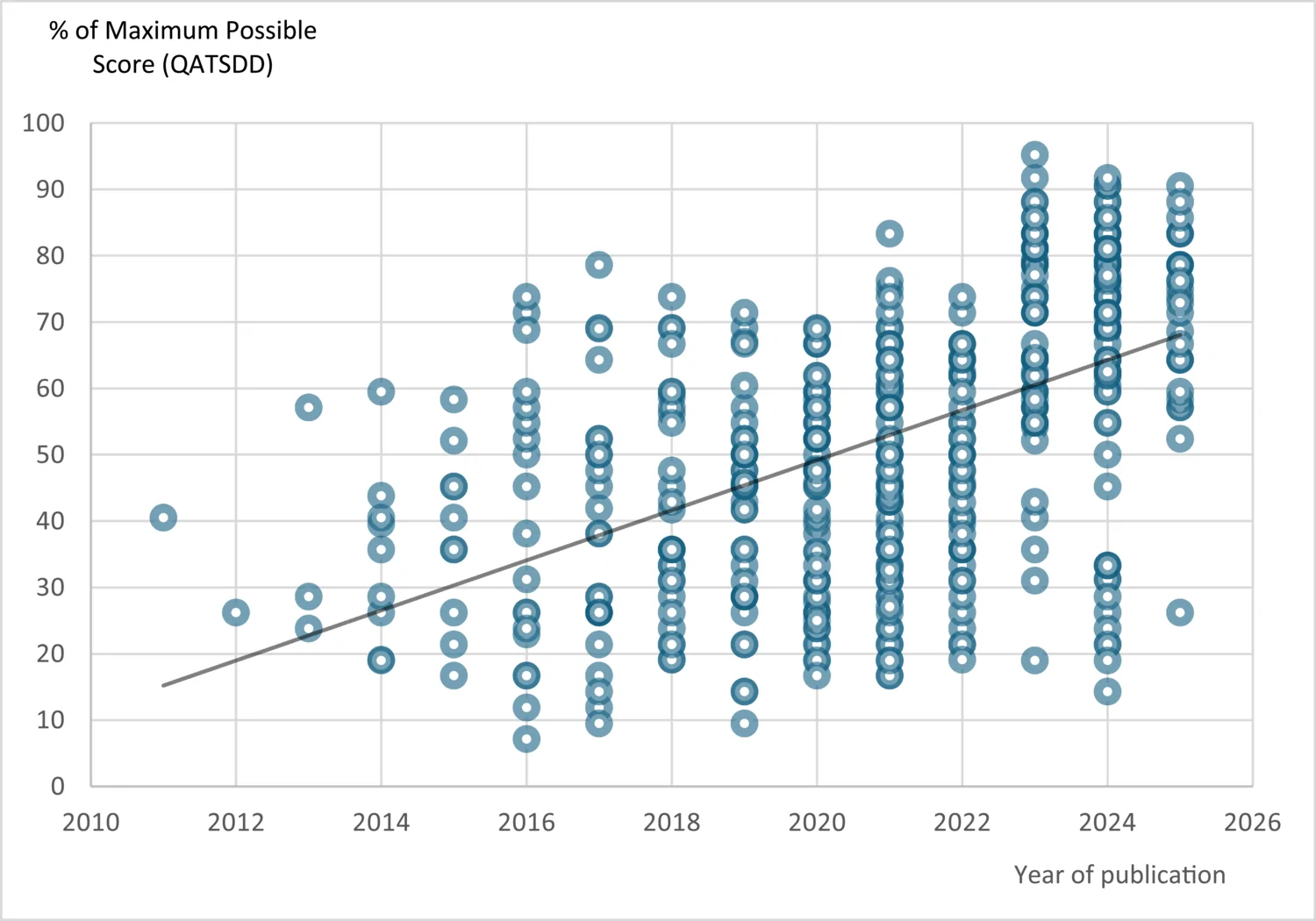
Quality assessment of studies using Quality Assessment Tool for Studies with Diverse Designs (QATSDD) tool per year of publication. It shows an increasing mean of the percentage (%) of maximum possible QATSDD score for the studies included over the years. A moderate positive correlation was found between the % of maximum possible QATSDD score and the year of publication (R = 0.534, *P* < 0.001). The mean global % of maximum possible QATSDD score was 52.6% (SD 20.5%; Minimum 7.14%, Maximum 95.2%).

## Discussion

### Main issues

#### General issues

Since their introduction in 2005, EPAs have become increasingly prevalent across all stages of medical education, reflecting their versatility and growing significance in various educational settings, potentially helping to address some of most relevant issues related to the implementation of CBME.

Taking this into account, our findings extend the work of previous studies [20–29], but also offer a more comprehensive overview that underscores the continuum of medical education and the evolution of EPAs from 2005 to 2025. Despite the possibility of using several review designs [33–36] for synthesising evidence in medical education, we decided to conduct this systematic review in an effort to use explicit and reproductible methods for searching, selecting, analysing and synthesising evidence related to the use of EPAs in an aggregative way.

Unlike the results presented by others [20], our review made it possible to identify a variety of educational frameworks associated with EPAs in a substantial number of the studies included. Examples of those key educational frameworks explored were the CBME [88], the AAMC core EPAs [148], the ACGME Core Competencies [253] and the CanMEDs [74]. In our review the accuracy of the nomenclature used was not explored following a systematic methodology. Nevertheless an after-analysis discussion made by our authors made it possible to identify some inconsistencies and to support the conclusions pointed out by other authors [20,21] considering the importance for a shared nomenclature to be clarified and applied in future studies by those dedicated to the use of EPAs.

#### Characteristics of the studies

Firstly, the results presented in our review focused on the exploration of the main characteristics of the studies included highlighting several relevant issues.

Similar to earlier observations [21,26], studies from Asia, Africa, the Middle East and South America were underrepresented, and PGME and some specialities were overrepresented in our review. Taken together these results have the potential of limiting the generalisability of our findings and call for future research to focus on the underrepresented contexts.

In contrast to other reviews [25–29], our study reported on the entire continuum of medical education and covered various specialties, offering a broader perspective on the application of EPAs.

Our review presents a variety of study designs and methodologies reported, making the extraction and analysis of data challenging. Such diversity can be a reflex of flexibility and adaptability of the strategies adopted to the particularities of the contexts studied. Nevertheless, such diversity can also present significant challenges for the interpretation of the results and may difficult the dissemination of the use of EPAs, as it hinders the definition of best practices.

The lack of interventional controlled trials may reflect the initial stages of the adoption of this framework and deserve additional attention in future research.

Many studies were conducted in single-centre settings and with small sample sizes, limiting the strength and generalisability of the conclusions drawn, as was also referred in other studies [22].

Unlike previous reviews [21,22,26], we included studies published in languages other than English. Despite this, most studies were still published in English, as was also observed by others [25], raising potential questions about the international representativeness of the findings, but also about the potential lack of relevance of this issue.

In contrast to patterns identified by other authors [21,22,27,29], our review revealed a shift towards studies focusing more on the implementation rather than the development of EPAs, suggesting a potential maturing in the research landscape. Despite the potential maturation referred, given the various challenges reported on the development, assessment, and impact assessment of the use of EPAs, we consider it advisable to continue the research focused on these issues.

#### Assessment of the impact of the use of EPAs

Secondly, we explored the assessment of the impact of the use of EPAs.

Reflecting a strong interest similar to that revealed by other authors [22], our review identified a significant number of studies evaluating the impact of the implementation of EPAs [50,202,204].

Despite the challenges associated with the evaluation of the costs of medical education [518], their particularities and relevance justify the attention given to this subject [519]. Notably, few studies in our review have reported on the cost-effectiveness of the educational interventions, exposing a possible research gap.

As other authors have noted [26], our review also highlights the possible influence of EPAs use on the improvement of the learning and assessment processes.

The frequent identification of gaps related to the validity of assessments, highlights a critical area that needs more robust research to better understand the impact of the use of EPAs, as similarly emphasised by other authors [29].

#### Development of EPAs

Thirdly, our work focused on the methodologies used for the development of EPAs.

Consistent with other researchers’ findings [22,28,29], our review reveals a substantial diversity in the strategies used to develop EPAs namely consensus methods [130,303], focus group techniques [65,190] and literature review [128,197]. This diversity potentially creates difficulties on the definition of best practices but may also indicate valuable adaptability to different contexts.

Our results reveal significant involvement of students/residents and patients/patient representatives during the development process, in contrast to earlier research [22,25]. This shift may reflect efforts to improve the involvement of key stakeholders in the development and implementation of EPAs.

#### Implementation of EPAs

Fourthly, we explored the methodologies used for the implementation of EPAs.

Our review emphasises the logistical difficulties (namely time, administrative support, costs and software support) [141,149,262,341], and faculty development challenges (particularly faculty development programme design and resources issues) [120,244,262] impacting EPAs’ implementation, which are essential for those starting to use EPAs frameworks and mirror challenges found in other studies [21,26,28].

In most of the studies, we found no reference to the use of development strategies for the different stakeholders, particularly faculty members. Given the importance of preparing the different stakeholders also highlighted previously [21,28], this finding may endanger the correct development and implementation of the EPAs framework, as noted by other authors [26] and deserves future research efforts.

#### Assessment of EPAs

Fifthly, our work reflected on several issues related to the assessment of EPAs.

The workplace-based evaluation of practice is the most commonly referred methodology referred for the assessment of EPAs [263,271].

Despite some scepticism about the validity of the use simulation-based data for making entrustment decisions [23], our review notes frequent references to the use of such strategies [123,326], suggesting an enduring interest and potential growth area.

The use of technological media for recording assessment and entrustment data has been noted in prior reviews [23,25] and, while our results support its widespread use showing the use of both website-based platforms [48,271] and mobile app-based platforms [225,338], most publications still do not mention the use of these kinds of resources.

As reported in other studies [23], our review also confirms the use of case-based discussions and product evaluations for entrustment decisions [186,210,226,341]. In contrast, the continued reference to written examinations in our findings [80,290] deviates from some recent trends [23], indicating a broader spectrum of assessment practices.

Many studies in our review report employing multiple methodologies for EPAs assessment, conducted at various times [103,262] and by different evaluators namely faculty members, self and peers evaluation [169,202,285]. Some authors [23] support this multifaceted approach, which can be considered an advisable practice for developing strong assessment frameworks.

The involvement of students and residents in the assessment process is still limited, which raises concerns that have also been reported by previous authors [26] and highlights difficulties in making the switch to a more learner-centred evaluation paradigm.

Various assessment tools were explored in the studies included namely global entrustment scales [196,232] and standardised rubrics [205,260]. It was noted that a small number of studies in our review reported rigorous validation efforts of the assessment tools used and this has also been reported by other authors [28], underscoring a significant area for future research.

The exploration of various assessment strategies is emphasised as necessary in our review, a conclusion supported by other authors [28].

#### Quality of the studies

As concluded by other authors [520], balancing methodological rigour with practical success can be challenging, but researchers developing EPAs must prioritise the quality of their approach. Taking this into consideration, the quality of the studies included, and the use of quality assessment tools and strategies were also explored in our review.

The studies included had an acceptable average quality, which was consistent with findings from other authors [23]. Our detailed analysis revealed specific items that future research should focus on improving. Our review also exposed a moderate positive association, between the quality of the included studies and the year of publication which supports the findings of other authors [25,29], revealing an apparent improvement of the quality of the studies over the years.

The lack of use of quality evaluation procedures and tools was highlighted in our review and may endanger the development and application of EPAs, a problem that was reported in previous studies [20,28,29].

Important methodological limitations were found in the studies included in our review, as was also highlighted in previous studies [21,27], emphasising the need for more rigorous methodological approaches in future studies focused on EPAs.

### Limitations of the systematic review

This review has some limitations that must be considered:

1. Scope and breadth of review: Given the heterogeneity of the included studies, a meta-analysis was not feasible, and our review presented a more exploratory character than initially expected. Nevertheless, the broadness of the studies included improved the generalisability of the findings across the medical education continuum.
2. Diversity of studies: The wide range of study types and educational stages of EPAs focused on our review made the data collection, analysis, and codification more difficult. However, by offering a thorough viewpoint on EPA usage in many contexts, this diversity also enriched our review.
3. Potential publication and selection biases:

- Our search strategy might have missed some important studies, given that we did not include individual term analyses within the context of ‘entrustable professional activities’, and because we limited our attention to studies that clearly focused on EPAs. Nonetheless, this decision increased the feasibility of our research.
- We might have missed some important articles because we didn't include grey literature in our review. We justify this option with the intent that our results reflect data from studies previously submitted to rigorous prepublication review reducing the risks of bias and inaccuracies, which cannot be guaranteed in all grey literature. In addition, we incorporated studies from six distinct electronic databases to increase the inclusiveness of potential studies.
- The exclusion of studies not published in English, French, Spanish or Portuguese may have caused the omission of some important studies published in other languages. Nevertheless, our results suggest that the impact of this choice is probably very low as most of the studies included were published in English independently of the context they referred to.
- It is possible that relevant studies were published after our search and were not included in our results. To reduce this limitation, we updated the records initially identified, including studies published until March 2025, and intend to further complement this review in the near future.

4. Quality assessment: The use of the QATSDD tool made it possible to conduct a workable quality evaluation across a variety of studies. Nevertheless, it may not have been as accurate as other instruments designed for particular study types. Additionally, an updated version of QATSDD tool was published improving some of the issues relating to its usability and clarity [521], however we were unable to use this new version as the design and implementation of our review had already commenced at the time of its publication.

5. EPA quality analysis: The quality of the EPAs reported in our review was not specifically examined. This analysis could reveal more insightful information, and future research should consider exploring this aspect

### Implications for practice and research

Our review is expected to have future implications both for practice and research related to EPAs:

1. Assessment of impact: A significant number of the studies included underscore the need to evaluate the impact of the use of EPAs not only on learner-centred and educational outcomes [214] but also on patient-centred outcomes [148,244], on key outcomes for the health systems [244], as well as the resources needed for the implementation of EPAs [214]. Following the efforts of previous authors [522], we also recommend that future research across the medical education continuum should better explore outcomes relevant to the various stakeholders, as this could help tailor EPAs more effectively in different educational contexts.
2. Methodological improvements: The results of our review emphasise the need for strategies that help improve the validity and generalisability of the results namely by exploring various research contexts in multicentric studies and by using validated tools to assess the quality of the EPAs implemented [46,98,149,187,248,271,296]. Researchers should prioritise the expansion of study contexts and populations, particularly in countries and specialities that are underrepresented, and additional caution and flexibility is advised when developing and implementing EPAs in those underrepresented environments. To promote the dissemination of EPAs in those contexts we suggest the implementation of faculty development programmes that include both stakeholders from those settings, and EPAs experts from environments where EPAs are already implemented. Such efforts are also expected to result in the improvement of the diversity of the research landscape while preserving its quality.
3. Challenges and best practices: Future studies should address the practical challenges encountered in the development, implementation, and assessment of EPAs, illustrated by the need to improve the impact evaluation of the use of EPAs highlighted in several studies included in our review [126,189,312]. To help overcome those difficulties, the adoption of implementation science principles [523], sustainable stakeholders’ development programmes [524], and various technological resources [525] can be particularly useful. Despite the challenges created by the diversity of study designs and methodologies reported in the studies included, our review presents results that also have the potential to inform future research aimed at identifying best practices for EPAs use.
4. Entrustment decisions: Several studies explored in our review also emphasise the need for future research to investigate the process of making entrustment decisions, including challenges, and influencing factors [126,131,174,256,259].
5. Resource utilisation: Numerous studies included highlight that the adoption of EPAs requires the use of a significant variety of resources ranging from dedicated time to software support [141,149,262,341]. Consequently, researchers should focus on evaluating the effects of the use of these resources to maximise the utilisation of EPAs.
6. Studies and EPAs quality assessment: To maintain high standards, it is important that future research establish standards for quality assessments utilising suitable tools. As such, a dedicated review centred on the quality of EPAs is suggested.
7. Comparative studies: As explored by other authors [526,527] the conduction and interpretation of interventional studies can be particularly challenging in the context of medical education given their complexity and the difficulty to isolate the effects of educational interventions. Nevertheless, echoing the conclusions of several studies analysed in our review [304,328], we also recommend conducting interventional studies that compare EPAs with other educational frameworks in order to demonstrate the benefits of EPAs and promote their broader recognition.
8. Advanced phase research: Given that most studies concentrate on the early stages of the development and implementation of EPAs, there is a need for research focused on the more advanced stages.
9. Clarification of concepts: The variability in the use and understanding of terms related to EPAs calls for studies that clarify and standardise nomenclature and concepts, ensuring uniformity and clear communication in medical education research.

## Conclusion

Since its introduction, EPAs have gained significant importance in the context of medical education, making competence-based medical education a closer and more practical reality.

Our review highlights the important evolution of EPAs, with an increasing volume of studies reporting on various aspects of their development, implementation, and assessment.

The attention given to several methodological issues, and the broader view brought by our review, contributes for the improvement in the understanding and application of EPAs, adding up to the knowledge presented by other authors.

Despite the interest revealed for this educational framework, several questions remain unanswered, and important challenges continue to be faced to improve the usage of EPAs.

Finally, our review emphasises research areas that deserve special attention in the near future.

## Supplementary Material

Supplement 2_Included Studies Details_EPA.xlsx Supplement 2_Included Studies Details_EPA.xlsx

Supplement 3_Journal of publication and Quartile analysis_EPA.docx Supplement 3_Journal of publication and Quartile analysis_EPA.docx

Supplement 4_Quality assessment using QATSDD_EPA.docx Supplement 4_Quality assessment using QATSDD_EPA.docx

Supplementary Material Supplement 1_Search strategy_EPA.docx

## Data Availability

Data is provided within the manuscript or supplementary information files. The datasets used and analysed in this study are available from the corresponding author on reasonable request.

## Systematic review registration

This systematic review was registered in the PROSPERO platform.

## References

[1] Custers E , Cate OT . The history of medical education in Europe and the United States, with respect to time and proficiency. Acad Med. 2018;93(3S Competency-Based, Time-Variable Education in the Health Professions):S49–S54. 10.1097/ACM.0000000000002079 29485488

[2] Irby DM , Cooke M , O'Brien BC . Calls for reform of medical education by the carnegie foundation for the advancement of teaching: 1910 and 2010. Acad Med. 2010;85(2):220–227. doi: 10.1097/ACM.0b013e3181c88449 20107346

[3] Kuper A , Whitehead C , Hodges BD . Looking back to move forward: using history, discourse and text in medical education research: AMEE guide no. 73. Med Teach. 2013;35(1):e849–60. doi: 10.3109/0142159X.2012.748887 23259609

[4] Carraccio C , Wolfsthal SD , Englander R , et al. Shifting paradigms: from flexner to competencies. Acad Med. 2002;77(5):361–367. doi: 10.1097/00001888-200205000-00003 12010689

[5] Hsiao CT , Chou FC , Hsieh CC , et al. Developing a competency-based learning and assessment system for residency training: analysis study of user requirements and acceptance. J Med Internet Res. 2020;22(4):e15655. doi: 10.2196/15655 PMC7189253 32286233

[6] Miller GE . The assessment of clinical skills/competence/performance. Acad Med. 1990;65(9 Suppl):S63–7. doi: 10.1097/00001888-199009000-00045 2400509

[7] Carraccio CL , Englander R . From flexner to competencies: reflections on a decade and the journey ahead. Acad Med. 2013;88(8):1067–1073. doi: 10.1097/ACM.0b013e318299396f 23807096

[8] Ten Cate O . Competency-based postgraduate medical education: past, present and future. GMS J Med Educ. 2017;34(5):Doc69. 10.3205/zma001146 PMC5704607 29226237

[9] Touchie C , ten Cate O . The promise, perils, problems and progress of competency-based medical education. Med Educ. 2016;50(1):93–100. doi: 10.1111/medu.12839 26695469

[10] Frank JR , Snell LS , Cate OT , et al. Competency-based medical education: theory to practice. Med Teach. 2010;32(8):638–645. doi: 10.3109/0142159X.2010.501190 20662574

[11] van Loon KA , Driessen EW , Teunissen PW , et al. Experiences with EPAs, potential benefits and pitfalls. Med Teach. 2014;36(8):698–702. doi: 10.3109/0142159X.2014.909588 24804911

[12] ten Cate O , Scheele F . Competency-based postgraduate training: can we bridge the gap between theory and clinical practice? Acad Med. 2007;82(6):542–547. doi: 10.1097/ACM.0b013e31805559c7 17525536

[13] Ten Cate O , Taylor DR . The recommended description of an entrustable professional activity: AMEE guide no. 140. Med Teach. 2020;43:1–9. doi: 10.1080/0142159X.2020.1838465 33167763

[14] ten Cate O . Entrustability of professional activities and competency-based training. Med Educ. 2005;39(12):1176–1177. doi: 10.1111/j.1365-2929.2005.02341.x 16313574

[15] Ten Cate O . Nuts and bolts of entrustable professional activities. J Grad Med Educ. 2013;5(1):157–158. doi: 10.4300/JGME-D-12-00380.1 PMC3613304 24404246

[16] Elnicki DM , Aiyer MK , Cannarozzi ML , et al. An entrustable professional activity (EPA)-Based framework to prepare fourth-year medical students for internal Medicine careers. J Gen Intern Med. 2017;32(11):1255–1260. doi: 10.1007/s11606-017-4089-8 PMC5653547 28634908

[17] Lomis K , Amiel JM , Ryan MS , et al. Implementing an entrustable professional activities framework in undergraduate medical education: early lessons from the AAMC core entrustable professional activities for entering residency pilot. Acad Med. 2017;92(6):765–770. doi: 10.1097/ACM.0000000000001543 28557937

[18] Jarrett JB , Antoun J , Hasnain M . Entrustable professional activity utilization: a CERA study of family Medicine residency program directors. Fam Med. 2019;51(6):471–476. doi: 10.22454/FamMed.2019.876961 31013347

[19] Broussenko M , Burns S , Leung FH , et al. Can teachers distinguish competencies from entrustable professional activities? PRiMER. 2019;3:19. doi: 10.22454/PRiMER.2019.433736 PMC7205098 32537590

[20] Meyer EG , Chen HC , Uijtdehaage S , et al. Scoping review of entrustable professional activities in undergraduate medical education. Acad Med. 2019;94(7):1040–1049. doi: 10.1097/ACM.0000000000002735 30946134

[21] Shorey S , Lau TC , Lau ST , et al. Entrustable professional activities in health care education: a scoping review. Med Educ. 2019;53(8):766–777. doi: 10.1111/medu.13879 30945329

[22] Bramley AL , McKenna L . Entrustable professional activities in entry-level health professional education: a scoping review. Med Educ. 2021;55(9):1011–1032. doi: 10.1111/medu.14539 33884655

[23] Duijn C , Dijk EJV , Mandoki M , et al. Assessment tools for feedback and entrustment decisions in the clinical workplace: a systematic review. J Vet Med Educ. 2019;46(3):340–352. doi: 10.3138/jvme.0917-123r 31460844

[24] Hennus MP , van Dam M , Gauthier S , et al. The logic behind entrustable professional activity frameworks: a scoping review of the literature. Med Educ. 2022;56(9):881–891. doi: 10.1111/medu.14806 PMC9542438 35388517

[25] Kerth JL , van Treel L , Bosse HM . The use of entrustable professional activities in pediatric postgraduate medical education: a systematic review. Acad Pediatr. 2022;22(1):21–28. doi: 10.1016/j.acap.2021.07.007 34256178

[26] Liu L , Jiang Z , Qi X , et al. An update on current EPAs in graduate medical education: a scoping review. Med Educ Online. 2021;26(1):1981198. doi: 10.1080/10872981.2021.1981198 PMC8477952 34569433

[27] Pinilla S , Lenouvel E , Strik W , et al. Entrustable professional activities in psychiatry: a systematic review. Acad Psychiatry. 2020;44(1):37–45. doi: 10.1007/s40596-019-01142-7 31732885

[28] Pinilla S , Lenouvel E , Cantisani A , et al. Working with entrustable professional activities in clinical education in undergraduate medical education: a scoping review. BMC Med Educ. 2021;21(1):172. doi: 10.1186/s12909-021-02608-9 PMC7980680 33740970

[29] O'Dowd E , Lydon S , O'Connor P , et al. A systematic review of 7 years of research on entrustable professional activities in graduate medical education, 2011-2018. Med Educ. 2019;53(3):234–249. doi: 10.1111/medu.13792 30609093

[30] Shea BJ , Grimshaw JM , Wells GA , et al. Development of AMSTAR: a measurement tool to assess the methodological quality of systematic reviews. BMC Med Res Methodol. 2007;7:10. doi: 10.1186/1471-2288-7-10 PMC1810543 17302989

[31] Merlin T , Weston A , Tooher R . Extending an evidence hierarchy to include topics other than treatment: revising The Australian 'levels of evidence. BMC Med Res Methodol. 2009;9:34. doi: 10.1186/1471-2288-9-34 PMC2700132 19519887

[32] Oxman AD , Guyatt GH . The science of reviewing research. Ann N Y Acad Sci. 1993;703:125–133. discussion 33-4. 10.1111/j.1749-6632.1993.tb26342.x 8192290

[33] Parker R , Sikora L . Literature reviews: key considerations and tips from knowledge synthesis librarians. J Grad Med Educ. 2022;14(1):32–35. doi: 10.4300/JGME-D-21-01114.1 PMC8848885 35222818

[34] Grant MJ , Booth A . A typology of reviews: an analysis of 14 review types and associated methodologies. Health Info Libr J. 2009;26(2):91–108. doi: 10.1111/j.1471-1842.2009.00848.x 19490148

[35] Gough D , Thomas J , Oliver S . Clarifying differences between review designs and methods. Syst Rev. 2012;1:28. doi: 10.1186/2046-4053-1-28 PMC3533815 22681772

[36] Gough D . Meta-narrative and realist reviews: guidance, rules, publication standards and quality appraisal. BMC Med. 2013;11:22. doi: 10.1186/1741-7015-11-22 PMC3606472 23360691

[37] Liberati A , Altman DG , Tetzlaff J , et al. The PRISMA statement for reporting systematic reviews and meta-analyses of studies that evaluate health care interventions: explanation and elaboration. J Clin Epidemiol. 2009;62(10):e1–34. doi: 10.1016/j.jclinepi.2009.06.006 19631507

[38] Ouzzani M , Hammady H , Fedorowicz Z , et al. Rayyan-a web and mobile app for systematic reviews. Syst Rev. 2016;5(1):210. doi: 10.1186/s13643-016-0384-4 PMC5139140 27919275

[39] Microsoft Microsoft® Excel® 2312 Build 16. 0. 17126. 20126 ed2025

[40] Sirriyeh R , Lawton R , Gardner P , et al. Reviewing studies with diverse designs: the development and evaluation of a new tool. J Eval Clin Pract. 2012;18(4):746–752. doi: 10.1111/j.1365-2753.2011.01662.x 21410846

[41] IBM . IBM SPSS Statistics. 29.0.0.0 (241) ed2024.

[42] OpenAI . ChatGPT[Large language model]. 2024. https://chat.openai.com/chat

[43] Learneo . QuillBot. 2024. https://quillbot.com/blog/

[44] Brasel KJ , Klingensmith ME , Englander R , et al. Entrustable professional activities in general surgery: development and implementation. J Surg Educ. 2019;76(5):1174–1186. doi: 10.1016/j.jsurg.2019.04.003 31029575

[45] Brown DR , Moeller JJ , Grbic D , et al. Entrustment decision making in the core entrustable professional activities: results of a multi-institutional study. Acad Med. 2022;97(4):536–543. doi: 10.1097/ACM.0000000000004242 34261864

[46] Chan B , Englander H , Kent K , et al. Transitioning toward competency: a resident-faculty collaborative approach to developing a transitions of care EPA in an internal Medicine residency program. J Grad Med Educ. 2014;6(4):760–764. doi: 10.4300/JGME-D-13-00414.1 PMC4477578 26140133

[47] Cutrer WB , Russell RG , Davidson M , et al. Assessing medical student performance of entrustable professional activities: a mixed methods comparison of co-activity and supervisory scales. Med Teach. 2020;42(3):325–332. doi: 10.1080/0142159X.2019.1686135 31714166

[48] Kalet A , Zabar S , Szyld D , et al. A simulated "Night-onCall" to assess and address the readiness-for-internship of transitioning medical students. Adv Simul (Lond). 2017;2:13. doi: 10.1186/s41077-017-0046-1 PMC5806245 29450014

[49] Michael M , Griggs AC , Shields IH , et al. Improving handover competency in preclinical medical and health professions students: establishing the reliability and construct validity of an assessment instrument. BMC Med Educ. 2021;21(1):518. doi: 10.1186/s12909-021-02943-x PMC8487478 34600497

[50] Norbury JW , Faulk CE , Harrell KM , et al. Impact of a revised curriculum focusing on clinical neurology and musculoskeletal care on a required fourth-year medical student physical Medicine and rehabilitation clerkship. Rehabil Res Pract. 2016;2016:6197961. doi: 10.1155/2016/6197961 PMC5153487 28025624

[51] Sample S , Al Rimawi H , Berczi B , et al. Seeing potential opportunities for teaching (SPOT): evaluating a bundle of interventions to augment entrustable professional activity acquisition. AEM Educ Train. 2021;5(4):e10631. doi: 10.1002/aet2.10631 PMC8381386 34471797

[52] Solymos O , Snyman L , Condon E , et al. Moving beyond the technical skills and promoting professionalism-the experience of the college of anaesthesiologists of Ireland with incorporating the medical council eight domains of good professional practice into entrustable professional activities. Ir J Med Sci. 2020;189(4):1379–1389. doi: 10.1007/s11845-020-02216-4 32248341

[53] Tanaka A , Kondo T , Urushibara-Miyachi Y , et al. Development of entrustable professional activities for residents rotating nephrology department in a Japanese university hospital: a delphi study. BMJ Open. 2021;11(8):e047923. doi: 10.1136/bmjopen-2020-047923 PMC8340278 34348951

[54] Acai A , Cupido N , Weavers A , et al. Competence committees: the steep climb from concept to implementation. Med Educ. 2021;55(9):1067–1077. doi: 10.1111/medu.14585 34152027

[55] Albright JB , Meier AH , Ruangvoravat L , et al. Association between entrustable professional activities and milestones evaluations: real-time assessments correlate with semiannual reviews. J Surg Educ. 2020;77(6):e220–e8. doi: 10.1016/j.jsurg.2020.07.027 32747323

[56] Aljamal YN , Farley DR . Making EPAs a 59 minute objective measure for surgical trainees - A pilot study. Am J Surg. 2020;219(1):185–190. doi: 10.1016/j.amjsurg.2019.07.034 31376952

[57] Amare EM , Siebeck M , Sendekie TY , et al. Development of an entrustable professional activities (EPA) framework to inform surgical residency training programs in Ethiopia: a three-round national delphi method study. J Surg Educ. 2022;79(1):56–68. doi: 10.1016/j.jsurg.2021.06.023 34294572

[58] Amiel JM , Andriole DA , Biskobing DM , et al. Revisiting the core entrustable professional activities for entering residency. Acad Med. 2021;96(7S):S14–S21. doi: 10.1097/ACM.0000000000004088 34183597

[59] Anderson TN , Kaba A , Gros E , et al. A novel blended curriculum for communication of informed consent with surgical interns. J Grad Med Educ. 2021;13(3):411–416. doi: 10.4300/JGME-D-20-01057.1 PMC8207932 34178267

[60] Andler C , Daya S , Kowalek K , et al. E-ASSESS: creating an EPA assessment tool for structured simulated emergency scenarios. J Grad Med Educ. 2020;12(2):153–158. doi: 10.4300/JGME-D-19-00533.1 PMC7161329 32322347

[61] Angus SV , Vu TR , Willett LL , et al. Internal Medicine residency program Directors' views of the core entrustable professional activities for entering residency: an opportunity to enhance communication of competency along the continuum. Acad Med. 2017;92(6):785–791. doi: 10.1097/ACM.0000000000001419 28557944

[62] Aube-Peterkin M , Ehlebracht A , Petrella F . Feedback and formative assessment in competency by design: the experience of residents and supervisors within a urology training program. Can Urol Assoc J. 2022;17. doi: 10.5489/cuaj.8103 PMC10073525 36486176

[63] Aulet TH , Moore JS , Callas PW , et al. (En)trust me: validating an assessment rubric for documenting clinical encounters during a surgery clerkship clinical skills exam. Am J Surg. 2020;219(2):258–262. doi: 10.1016/j.amjsurg.2018.12.055 30639130

[64] Austin JP , Baskerville M , Bumsted T , et al. Development and evaluation of a simulation-based transition to clerkship course. Perspect Med Educ. 2020;9(6):379–384. doi: 10.1007/S40037-020-00590-4 PMC7718359 32458381

[65] Aylward M , Nixon J , Gladding S . An entrustable professional activity (EPA) for handoffs as a model for EPA assessment development. Acad Med. 2014;89(10):1335–1340. doi: 10.1097/ACM.0000000000000317 24892402

[66] Baer HR , Gilbert AR , Forster JE , et al. Use of the electrodiagnostic entrustable professional activity for competency assessment in physical Medicine and rehabilitation training programs. Am J Phys Med Rehabil. 2020;99(1):81–85. doi: 10.1097/PHM.0000000000001302 31464758

[67] Baghus A , Giroldi E , Muris J , et al. Identifying entrustable professional activities for shared decision making in postgraduate medical education: a national delphi study. Acad Med. 2021;96(1):126–133. doi: 10.1097/ACM.0000000000003618 PMC7769188 32739926

[68] Barakat LA , Dunne DW , Tetrault JM , et al. The changing face of HIV care: expanding HIV training in an internal Medicine residency program. Acad Med. 2018;93(11):1673–1678. doi: 10.1097/ACM.0000000000002317 29901657

[69] Benstead K , Lara PC , Andreopoulos D , et al. Recommended ESTRO core curriculum for radiation Oncology/Radiotherapy 4th edition. Radiother Oncol. 2019;141:1–4. doi: 10.1016/j.radonc.2019.08.013 31495514

[70] Benstead K , Lara PC , Eller Y , et al. Clinical oncology module for the ESTRO core curriculum. Radiother Oncol. 2021;156:19–22. doi: 10.1016/j.radonc.2020.11.029 33276018

[71] Berberat PO , Rotthoff T , Baerwald C , et al. Entrustable professional activities in final year undergraduate medical training - advancement of the final year training logbook in Germany. GMS J Med Educ. 2019;36(6):Doc70. 10.3205/zma001278 PMC6905355 31844642

[72] Bonnie LHA , Nasori M , Visser MRM , et al. Feasibility, and validity aspects of entrustable professional activity (EPA)-based assessment in general practice training. Educ Prim Care. 2022;33(2):69–76. doi: 10.1080/14739879.2021.1951127 34415820

[73] Bonnie LHA , Visser MRM , Bont J , et al. Trainers' and trainees' expectations of entrustable professional activities (EPAs) in a primary care training programme. Educ Prim Care. 2019;30(1):13–21. doi: 10.1080/14739879.2018.1532773 30526392

[74] Boyce P , Spratt C , Davies M , et al. Using entrustable professional activities to guide curriculum development in psychiatry training. BMC Med Educ. 2011;11:96. doi: 10.1186/1472-6920-11-96 PMC3295698 22112295

[75] Bradley EB , Waselewski EA , Gusic ME . How do clerkship students use EPA data? Illuminating Students' perspectives as partners in programs of assessment. Med Sci Educ. 2021;31(4):1419–1428. doi: 10.1007/s40670-021-01327-6 PMC8368261 34457983

[76] Branfield Day L , Miles A , Ginsburg S , et al. Resident perceptions of assessment and feedback in competency-based medical education: a focus group study of one internal Medicine residency program. Acad Med. 2020;95(11):1712–1717. doi: 10.1097/ACM.0000000000003315 32195692

[77] Bratz J , Bussenius L , Bratz I , et al. Assessment of final-year medical students' entrustable professional activities after education on an interprofessional training ward: a case-control study. Perspect Med Educ. 2022;11(5):266–272. doi: 10.1007/S40037-022-00720-0 PMC9302559 35864296

[78] Bray MJ , Bradley EB , Martindale JR , et al. Implementing systematic faculty development to support an EPA-Based program of assessment: strategies, outcomes, and lessons learned. Teach Learn Med. 2021;33(4):434–444. doi: 10.1080/10401334.2020.1857256 33331171

[79] Bremer AE , van de Pol MHJ , Laan R , et al. How an EPA-based curriculum supports professional identity formation. BMC Med Educ. 2022;22(1):48. doi: 10.1186/s12909-022-03116-0 PMC8781044 35057788

[80] Brenner J , Bird J , Ginzburg SB , et al. Trusting early learners with critical professional activities through emergency medical technician certification. Med Teach. 2018;40(6):561–568. doi: 10.1080/0142159X.2018.1444745 29519189

[81] Brown Jr. CR , Criscione-Schreiber L , O'Rourke KS , et al. What is a rheumatologist and how do we make one? Arthritis Care Res (Hoboken). 2016;68(8):1166–1172. doi: 10.1002/acr.22817 26663526

[82] Brown DR , Moeller JJ , Grbic D , et al. Comparing entrustment decision-making outcomes of the core entrustable professional activities pilot, 2019-2020. JAMA Netw Open. 2022;5(9):e2233342. doi: 10.1001/jamanetworkopen.2022.33342 PMC9513644 36156144

[83] Brown DR , Warren JB , Hyderi A , et al. Finding a path to entrustment in undergraduate medical education: a progress report from the AAMC core entrustable professional activities for entering residency entrustment concept group. Acad Med. 2017;92(6):774–779. doi: 10.1097/ACM.0000000000001544 28557941

[84] Bruce AN , Kumar A , Malekzadeh S . Procedural skills of the entrustable professional activities: are graduating US medical students prepared to perform procedures in residency? J Surg Educ. 2017;74(4):589–595. doi: 10.1016/j.jsurg.2017.01.002 28126380

[85] Bryant BH . Feasibility of an entrustable professional activity for pathology resident frozen section training. Acad Pathol. 2021;8:23742895211041757. doi: 10.1177/23742895211041757 PMC8411628 34485691

[86] Butani L , Plant J , Barone MA , et al. Entrustable professional activity-based assessments in undergraduate medical education: a survey of pediatric educators. Acad Pediatr. 2021;21(5):907–911. doi: 10.1016/j.acap.2021.03.020 33838345

[87] Bynum D , Colford C , Royal K . Teaching medical students the art of the 'write-up. Clin Teach. 2015;12(4):246–249. doi: 10.1111/tct.12304 26036763

[88] Calaman S , Hepps JH , Bismilla Z , et al. The creation of standard-setting videos to support faculty observations of learner performance and entrustment decisions. Acad Med. 2016;91(2):204–209. doi: 10.1097/ACM.0000000000000853 26266461

[89] Carey R , Wilson G , Bandi V , et al. Developing a dashboard to meet the needs of residents in a competency-based training program: a design-based research project. Can Med Educ J. 2020;11(6):e31–e45. 10.36834/cmej.69682 PMC7749685 33349752

[90] CarlLee S , Rowat J , Suneja M . Assessing entrustable professional activities using an orientation OSCE: identifying the gaps. J Grad Med Educ. 2019;11(2):214–220. doi: 10.4300/JGME-D-18-00601.2 PMC6476083 31024656

[91] Caro Monroig AM , Chen HC , Carraccio C , et al. Group ES. Medical Students' perspectives on entrustment decision making in an entrustable professional activity assessment framework: a secondary data analysis. Acad Med. 2021;96(8):1175–1181. doi: 10.1097/ACM.0000000000003858 33239536

[92] Carraccio C , Martini A , Van Melle E , et al. Identifying core components of EPA implementation: a path to knowing if a complex intervention is being implemented as intended. Acad Med. 2021;96(9):1332–1336. doi: 10.1097/ACM.0000000000004075 33769339

[93] Cavalcanti M , Fernandes AK , McCallister JW , et al. Post-clerkship curricular reform: specialty-specific tracks and entrustable professional activities to guide the transition to residency. Med Sci Educ. 2021;31(2):851–861. doi: 10.1007/s40670-021-01248-4 PMC7929549 33686361

[94] Caverzagie KJ , Cooney TG , Hemmer PA , et al. The development of entrustable professional activities for internal Medicine residency training: a report from the education redesign committee of the alliance for academic internal Medicine. Acad Med. 2015;90(4):479–484. doi: 10.1097/ACM.0000000000000564 25406600

[95] Cervantes-Sanchez CR , Parra-Acosta H , Cantu-Reyes JC . Diseno de un instrumento para evaluar las actividades profesionales confiables en cirugia general. Cir Cir. 2022;90(6):813–821. doi: 10.24875/CIRU.22000038 36472857

[96] Chamberlain NR , Sexton PS , Hardee MR , et al. Physician-mentored patient rounds to observe and assess entrustable professional activities 1 and 2 in preclinical medical students. J Am Osteopath Assoc. 2018;118(3):199–206. doi: 10.7556/jaoa.2018.039 29480920

[97] Chang A , Bowen JL , Buranosky RA , et al. Transforming primary care training--patient-centered medical home entrustable professional activities for internal Medicine residents. J Gen Intern Med. 2013;28(6):801–809. doi: 10.1007/s11606-012-2193-3 PMC3663955 22997002

[98] Chaplin T , Egan R , Cofie N , et al. The implementation of a multi-institutional multidisciplinary simulation-based resuscitation skills training curriculum. Cureus. 2018;10(11):e3593 10.7759/cureus.3593 PMC6338407 30680256

[99] Chauhan VS , Chatterjee K , Prakash J , et al. Identifying entrustable professional activities for postgraduation in psychiatry: what should a psychiatrist be able to do? Ind Psychiatry J. 2022;31(1):49–55. doi: 10.4103/ipj.ipj_124_21 PMC9255628 35800858

[100] Chen HC , McNamara M , Teherani A , et al. Developing entrustable professional activities for entry into clerkship. Acad Med. 2016;91(2):247–255. doi: 10.1097/ACM.0000000000000988 26556295

[101] Chen XP , Harzman A , Cochran A , et al. Evaluation of an instrument to assess resident surgical entrustable professional activities (SEPAs). Am J Surg. 2020;220(1):4–7. doi: 10.1016/j.amjsurg.2019.08.026 31526512

[102] Cheung K , Rogoza C , Chung AD , et al. Analyzing the administrative burden of competency based medical education. Can Assoc Radiol J. 2022;73(2):299–304. doi: 10.1177/08465371211038963 34449283

[103] Choe JH , Knight CL , Stiling R , et al. Shortening the miles to the milestones: connecting EPA-Based evaluations to ACGME milestone reports for internal Medicine residency programs. Acad Med. 2016;91(7):943–950. doi: 10.1097/ACM.0000000000001161 27028030

[104] Cohen BL , Gallinger ZR , Ha C , et al. Development of entrustable professional activities for advanced inflammatory bowel disease fellowship training in the United States. Inflamm Bowel Dis. 2020;26(9):1291–1305. doi: 10.1093/ibd/izaa177 32820340

[105] Colbert-Getz JM , Lappe K , Northrup M , et al. To what degree are the 13 entrustable professional activities already incorporated into Physicians' performance schemas for medical students? Teach Learn Med. 2019;31(4):361–369. doi: 10.1080/10401334.2019.1573146 30873878

[106] Collins E , Stahl C , Schnapp B , et al. Changing medical education when change is hard: implementing an interdepartmental entrustable professional activity. AEM Educ Train. 2021;5(3):e10561. doi: 10.1002/aet2.10561 PMC8171789 34124509

[107] Costich M , Finkel MA , Friedman S , et al. Transition-to-residency: pilot innovative, online case-based curriculum for medical students preparing for pediatric internships. Med Educ Online. 2021;26(1):1892569. doi: 10.1080/10872981.2021.1892569 PMC7906614 33618622

[108] Cotta CV , Ondrejka SL , Nakashima MO , et al. Pathology residents as testing personnel in the hematology laboratory. Arch Pathol Lab Med. 2022;146(7):894–902. doi: 10.5858/arpa.2020-0630-OA 34619751

[109] Crannell WC , Zakhary B , Hamilton H , et al. Design of an entrustable professional activity for adult extracorporeal membrane oxygenation. Surg Open Sci. 2020;2(1):42–45. doi: 10.1016/j.sopen.2019.09.001 PMC8083009 33981980

[110] Curran VR , Deacon D , Schulz H , et al. Evaluation of the characteristics of a workplace assessment form to assess entrustable professional activities (EPAs) in an undergraduate surgery core clerkship. J Surg Educ. 2018;75(5):1211–1222. doi: 10.1016/j.jsurg.2018.02.013 29609893

[111] Czeskleba A , Holzhausen Y , Peters H . Patient safety during final-year clerkships: a qualitative study of possible error sources and of the potential of entrustable professional activities. GMS J Med Educ. 2019;36(2):Doc18. 10.3205/zma001226 PMC6446462 30993176

[112] Datta K , Guru CS , Krishnan A , et al. Entrustable professional activities (EPAs) and milestones for MD sports Medicine: a proposed portfolio. Med J Armed Forces India. 2021;77(Suppl 1):S129–S33. 10.1016/j.mjafi.2020.12.026 PMC7873686 33612943

[113] Datta R , Datta K , Routh D , et al. Development of a portfolio framework for implementation of an outcomes-based healthcare professional education curriculum using a modified e-Delphi method. Med J Armed Forces India. 2021;77(Suppl 1):S49–S56. doi: 10.1016/j.mjafi.2020.11.012 PMC7873694 33612932

[114] Datta R , Raghavan D , Anand V , et al. Identifying entrustable professional activities for post-graduation in ENT: what should an ENT specialist be able to do? Med J Armed Forces India. 2021;77(Suppl 1):S168–S72. 10.1016/j.mjafi.2020.12.031 PMC7873687 33612949

[115] Dehghani Poudeh M , Mohammadi A , Mojtahedzadeh R , et al. Entrustability levels of general internal Medicine residents. BMC Med Educ. 2021;21(1):185. doi: 10.1186/s12909-021-02624-9 PMC7995576 33766005

[116] Deitte LA , Gordon LL , Zimmerman RD , et al. Entrustable professional activities: ten things radiologists do. Acad Radiol. 2016;23(3):374–381. doi: 10.1016/j.acra.2015.11.010 26781203

[117] Diwersi N , Gass JM , Fischer H , et al. Surgery goes EPA (Entrustable Professional Activity) - how a strikingly easy to use app revolutionizes assessments of clinical skills in surgical training. BMC Med Educ. 2022;22(1):559. doi: 10.1186/s12909-022-03622-1 PMC9295378 35854302

[118] Duggan N , Curran VR , Fairbridge NA , et al. Using mobile technology in assessment of entrustable professional activities in undergraduate medical education. Perspect Med Educ. 2021;10(6):373–377. doi: 10.1007/S40037-020-00618-9 PMC8633342 33095399

[119] Duijn C , Welink LS , Bok HGJ , et al. When to trust our learners? Clinical teachers' perceptions of decision variables in the entrustment process. Perspect Med Educ. 2018;7(3):192–199. doi: 10.1007/S40037-018-0430-0 PMC6002285 29713908

[120] Duijn C , Welink LS , Mandoki M , et al. Am I ready for it? Students' Perceptions of meaningful feedback on entrustable professional activities. Perspect Med Educ. 2017;6(4):256–264. doi: 10.1007/S40037-017-0361-1 PMC5542892 28577253

[121] Dunne D , Gielissen K , Slade M , et al. WBAs in UME-How many are needed? A reliability analysis of 5 AAMC core EPAs implemented in the internal Medicine clerkship. J Gen Intern Med. 2022;37(11):2684–2690. doi: 10.1007/s11606-021-07151-3 PMC9411433 34561828

[122] Dunne D , Green M , Tetrault J , et al. Development of a novel competency-based evaluation system for HIV primary care training: the HIV entrustable professional activities. J Gen Intern Med. 2020;35(1):331–335. doi: 10.1007/s11606-019-04956-1 PMC6957645 31667752

[123] Dwyer T , Wadey V , Archibald D , et al. Cognitive and psychomotor entrustable professional activities: can simulators help assess competency in trainees? Clin Orthop Relat Res. 2016;474(4):926–934. doi: 10.1007/s11999-015-4553-x PMC4773339 26394640

[124] El-Haddad C , Damodaran A , McNeil HP , et al. A patient-centered approach to developing entrustable professional activities. Acad Med. 2017;92(6):800–808. doi: 10.1097/ACM.0000000000001616 28557946

[125] El-Haddad C , Hegazi I , Hu W . A patient expectations questionnaire for determining criteria for entrustment decisions. Med Teach. 2021;43(9):1031–1038. doi: 10.1080/0142159X.2021.1907324 33840349

[126] Emke AR , Park YS , Srinivasan S , et al. Workplace-based assessments using pediatric critical care entrustable professional activities. J Grad Med Educ. 2019;11(4):430–438. doi: 10.4300/JGME-D-18-01006.1 PMC6699545 31440338

[127] Esquivel EL , De Angelis P , Chae JK , et al. Transitioning preclinical students into clerkships amidst curricular disruptions from the COVID-19 pandemic. Med Educ Online. 2021;26(1):1996216. doi: 10.1080/10872981.2021.1996216 PMC8555517 34710002

[128] Fazio SB , Ledford CH , Aronowitz PB , et al. Competency-based medical education in the internal Medicine clerkship: a report from the alliance for academic internal Medicine undergraduate medical education task force. Acad Med. 2018;93(3):421–427. doi: 10.1097/ACM.0000000000001896 28930762

[129] Fehr F , Weiss- Becker BC , Opladen H . T. Entrustable professional activities in post-licensure training in primary care pediatrics: necessity, development and implementation of a competency-based post-graduate curriculum. GMS J Med Educ. 2017;34(5):Doc67. 10.3205/zma001144 PMC5704614 29226235

[130] Fessler HE , Addrizzo-Harris D , Beck JM , et al. Entrustable professional activities and curricular milestones for fellowship training in pulmonary and critical care Medicine: report of a multisociety working group. Chest. 2014;146(3):813–834. doi: 10.1378/chest.14-0710 24945874

[131] Fincke F , Prediger S , Schick K , et al. Entrustable professional activities and facets of competence in a simulated workplace-based assessment for advanced medical students. Med Teach. 2020;42(9):1019–1026. doi: 10.1080/0142159X.2020.1779204 32579039

[132] Fisk D , Clendenning B , St John P , et al. Multi-stakeholder validation of entrustable professional activities for a family Medicine care of the elderly residency program: a focus group study. Gerontol Geriatr Educ. 2022;45:1–14. doi: 10.1080/02701960.2022.2130913 36326195

[133] Francischetti I , Holzhausen Y , Peters H . Entrustable professional activities for junior Brazilian medical students in community Medicine. BMC Med Educ. 2022;22(1):737. doi: 10.1186/s12909-022-03762-4 PMC9598029 36284283

[134] Gaffney S , Farnan JM , Hirsch K , et al. The modified, multi-patient observed simulated handoff experience (M-OSHE): assessment and feedback for entering residents on handoff performance. J Gen Intern Med. 2016;31(4):438–441. doi: 10.1007/s11606-016-3591-8 PMC4803693 26831306

[135] Gao S , Li N , Wang X , et al. An assessment framework for the training of general practitioners and specialists based on EPAs. Front Public Health. 2022;10:896097. doi: 10.3389/fpubh.2022.896097 PMC9300895 35875034

[136] Garofalo M , Aggarwal R . Obstetrics and gynecology modified delphi survey for entrustable professional activities: quantification of importance, benchmark levels, and roles in simulation-based training and assessment. Cureus. 2018;10(7):e3051. doi: 10.7759/cureus.3051 PMC6157652 30271696

[137] Gomes MM , Driman D , Park YS , et al. Teaching and assessing intra-operative consultations in competency-based medical education: development of a workplace-based assessment instrument. Virchows Arch. 2021;479(4):803–813. doi: 10.1007/s00428-021-03113-6 PMC8516791 33966099

[138] Gonzalo JD , Wolpaw DR , Krok KL , et al. A developmental approach to internal Medicine residency education: lessons learned from the design and implementation of a novel longitudinal coaching program. Med Educ Online. 2019;24(1):1591256. doi: 10.1080/10872981.2019.1591256 PMC6442085 30924404

[139] Graafland M , Ten Cate O , van Seventer JP , et al. Mapping the demand for serious games in postgraduate medical education using the entrustable professional activities framework. Games Health J. 2015;4(5):381–386. doi: 10.1089/g4h.2014.0129 26287929

[140] Grbic D , Gielissen KA , Obeso V , et al. Core entrustable professional activities for entering residency: a national survey of graduating medical Students' self-assessed skills by specialty. J Am Coll Surg. 2022;235(6):940–951. doi: 10.1097/XCS.0000000000000395 PMC9653107 36102502

[141] Gruppen LD , Burkhardt JC , Fitzgerald JT , et al. Competency-based education: programme design and challenges to implementation. Med Educ. 2016;50(5):532–539. doi: 10.1111/medu.12977 27072442

[142] Gunasingha RM , Knudsen N , Scialla T , et al. Vital conversations: an interactive conflict resolution training session for fourth-year medical students. MedEdPORTAL. 2021;17:11074. doi: 10.15766/mep_2374-8265.11074 PMC7830754 33511271

[143] Gupta A , Watkins AC , Fahey TJ , et al. Entrustable professional activities: do general surgery residents trust them? J Surg Educ. 2020;77(3):520–526. doi: 10.1016/j.jsurg.2019.12.005 31948866

[144] Hamui-Sutton A , Monterrosas-Rojas AM , Ortiz-Montalvo A , et al. Specific entrustable professional activities for undergraduate medical internships: a method compatible with the academic curriculum. BMC Med Educ. 2017;17(1):143. doi: 10.1186/s12909-017-0980-6 PMC5574123 28841876

[145] Hanson MN , Pryor AD , Jeyarajah DR , et al. Implementation of entrustable professional activities into fellowship council accredited programs: a pilot project. Surg Endosc. 2022;37:1–10. doi: 10.1007/s00464-022-09502-5 PMC9381155 35974253

[146] Hart D , Franzen D , Beeson M , et al. Integration of entrustable professional activities with the milestones for emergency Medicine residents. West J Emerg Med. 2019;20(1):35–42. 10.5811/westjem.2018.11.38912 PMC6324698 30643599

[147] Hasty BN , Lau JN , Tekian A , et al. Validity evidence for a knowledge assessment tool for a mastery learning scrub training curriculum. Acad Med. 2020;95(1):129–135. doi: 10.1097/ACM.0000000000003007 31577588

[148] Hauer KE , Boscardin C , Fulton TB , et al. Using a curricular vision to define entrustable professional activities for medical student assessment. J Gen Intern Med. 2015;30(9):1344–1348. doi: 10.1007/s11606-015-3264-z PMC4539334 26173516

[149] Hauer KE , Soni K , Cornett P , et al. Developing entrustable professional activities as the basis for assessment of competence in an internal Medicine residency: a feasibility study. J Gen Intern Med. 2013;28(8):1110–1114. doi: 10.1007/s11606-013-2372-x PMC3710378 23595926

[150] Heimroth J , Jones VM , Howard Jr. JD , et al. Utilizing surgical bootcamps to teach core entrustable professional activities to senior medical students. Am Surg. 2018;84(6):783–788. doi: 10.1177/000313481808400619 29981602

[151] Hennus MP , Nusmeier A , van Heesch GGM , et al. Development of entrustable professional activities for paediatric intensive care fellows: a national modified delphi study. PLoS One. 2021;16(3):e0248565. doi: 10.1371/journal.pone.0248565 PMC7971696 33735195

[152] Herath JC . Firsthand experience in graduating three cohorts of forensic pathologists trained with competency by design (CBD) curriculum. Acad Forensic Pathol. 2021;11(4):196–207. doi: 10.1177/19253621211063104 PMC8727842 35003451

[153] Herrigel DJ , Donovan C , Goodman E , et al. Simulation as a platform for development of entrustable professional activities: a modular, longitudinal approach. Cureus. 2020;12(10):e11098 10.7759/cureus.11098 PMC7681749 33240694

[154] Hmoud AlSheikh M , Zaini RG , Iqbal MZ . Developing and mapping entrustable professional activities with Saudi meds competency framework: a consensus study. Adv Med Educ Pract. 2022;13:1367–1374. doi: 10.2147/AMEP.S379184 PMC9624166 36330463

[155] Hobday PM , Borman-Shoap E , Cullen MJ , et al. The minnesota method: a learner-driven, entrustable professional activity-based comprehensive program of assessment for medical students. Acad Med. 2021;96(7S):S50–S5. 10.1097/ACM.0000000000004101 34183602

[156] Holmstrom AL , Chia MC , O'Brien CL , et al. Entrustable professional activity-based summative performance assessment in the surgery clerkship. J Surg Educ. 2021;78(4):1144–1150. doi: 10.1016/j.jsurg.2020.12.001 33384267

[157] Holzhausen Y , Maaz A , Renz A , et al. Development of entrustable professional activities for entry into residency at the charite Berlin. GMS J Med Educ. 2019;36(1):Doc5 10.3205/zma001213 PMC6390089 30828605

[158] Holzhausen Y , Maaz A , Roa-Romero Y , et al. What can we expect from medical graduates? Empirical survey on the performance of core EPAs in the first days of residency. BMC Med Educ. 2020;20(1):452. doi: 10.1186/s12909-020-02376-y PMC7685603 33228704

[159] Hsu D , Nypaver M , Fein DM , et al. Essentials of PEM fellowship part 2: the profession in entrustable professional activities. Pediatr Emerg Care. 2016;32(6):410–418. doi: 10.1097/PEC.0000000000000827 27253361

[160] Huth K , Henry D , Cribb Fabersunne C , et al. A multistakeholder approach to the development of entrustable professional activities in complex care. Acad Pediatr. 2022;22(2):184–189. doi: 10.1016/j.acap.2021.09.014 34600114

[161] Huth K , Henry D , Cribb Fabersunne C , et al. Family-educator partnership in the development of entrustable professional activities in complex care. Acad Med. 2022;98:342–347. doi: 10.1097/ACM.0000000000005095 36512821

[162] Iqbal MZ , Konings KD , Al-Eraky M , et al. Development of an entrustable professional activities (EPAs) framework for small group facilitators through a participatory design approach. Med Educ Online. 2020;25(1):1694309. doi: 10.1080/10872981.2019.1694309 PMC6968595 31876251

[163] Iqbal MZ , Konings KD , Al-Eraky MM , et al. Entrustable professional activities for small-group facilitation: a validation study using modified delphi technique. Teach Learn Med. 2021;33(5):536–545. doi: 10.1080/10401334.2021.1877714 33588650

[164] Johnson NR , Pelletier A , Berkowitz LR . Mini-clinical evaluation exercise in the era of milestones and entrustable professional activities in obstetrics and gynaecology: resume or reform? J Obstet Gynaecol Can. 2020;42(6):718–725. doi: 10.1016/j.jogc.2019.10.002 31882285

[165] Jonker G , Booij E , Otte WR , et al. An elective entrustable professional activity-based thematic final medical school year: an appreciative inquiry study among students, graduates, and supervisors. Adv Med Educ Pract. 2018;9:837–845. doi: 10.2147/AMEP.S176649 PMC6241681 30532611

[166] Jonker G , Hoff RG , Max S , et al. Connecting undergraduate and postgraduate medical education through an elective EPA-based transitional year in acute care: an early project report. GMS J Med Educ. 2017;34(5):Doc64 10.3205/zma001141 PMC5704612 29226232

[167] Kamp MA , Malzkorn B , von Sass C , et al. Proposed definition of competencies for surgical neuro-oncology training. J Neurooncol. 2021;153(1):121–131. doi: 10.1007/s11060-021-03750-6 PMC8131302 33881726

[168] Kane SK , Lorant DE . Creation and validation of tool to assess resident competence in neonatal resuscitation. Acad Pediatr. 2019;19(4):394–398. doi: 10.1016/j.acap.2018.09.004 30273690

[169] Kang AJ , Gielissen K , Windish D . Using an entrustable professional activity to assess consultation requests called on an internal Medicine teaching service. MedEdPORTAL. 2019;15:10854. doi: 10.15766/mep_2374-8265.10854 PMC6953740 31934616

[170] Karthikeyan P , Pulimoottil DT . Design and implementation of competency based postgraduate medical education in otorhinolaryngology: the pilot experience in India. Indian J Otolaryngol Head Neck Surg. 2019;71(Suppl 1):671–678. doi: 10.1007/s12070-018-1474-5 PMC6848633 31742040

[171] Katz SJ , Wang D . Comparing entrustable professional activity scores given by faculty physicians and senior trainees to first-year residents. Cureus. 2022;14(6):e25798. doi: 10.7759/cureus.25798 PMC9273189 35836450

[172] Kearse LE , Schmiederer IS , Anderson TN , et al. American board of surgery entrustable professional activities (EPAs): assessing graduating Residents' perception of preoperative entrustment. J Surg Educ. 2021;78(6):e183–e8. doi: 10.1016/j.jsurg.2021.09.004 34602378

[173] Keeley MG , Gusic ME , Morgan HK , et al. Moving toward summative competency assessment to individualize the postclerkship phase. Acad Med. 2019;94(12):1858–1864. doi: 10.1097/ACM.0000000000002830 31169542

[174] Kelleher M , Kinnear B , Sall D , et al. A reliability analysis of entrustment-derived workplace-based assessments. Acad Med. 2020;95(4):616–622. doi: 10.1097/ACM.0000000000002997 31567170

[175] Kemp WL , Koponen M , Sens MA . Forensic autopsy experience and core entrustable professional activities: a structured introduction to autopsy pathology for preclinical student. Acad Pathol. 2019;6:2374289519831930. doi: 10.1177/2374289519831930 PMC6402054 30859125

[176] Kessoku T , Uneno Y , Urushibara-Miyachi Y , et al. Development of a list of competencies and entrustable professional activities for resident physicians during death pronouncement: a modified delphi study. BMC Med Educ. 2022;22(1):119. doi: 10.1186/s12909-022-03149-5 PMC8861606 35193555

[177] Kim C , Lin S , Sattler AL . A model medical student-led interprofessional QI project on lab monitoring. PRiMER. 2018;2:13. doi: 10.22454/PRiMER.2018.187050 PMC7426132 32818186

[178] Klapheke M , Johnson T , Cubero M . Assessing entrustable professional activities during the psychiatry clerkship. Acad Psychiatry. 2017;41(3):345–349. doi: 10.1007/s40596-017-0665-9 28315194

[179] Ko TW , Hung SH , Wu YL , et al. Can simulation-based team resource management training help students to pass entrustable professional activities 13? BMJ Simul Technol Enhanc Learn. 2020;6(1):22–25. doi: 10.1136/bmjstel-2018-000397 PMC8936791 35514457

[180] Kouzmina E , Mann S , Chaplin T , et al. An evaluation of the surgical foundations curriculum: a national study. J Surg Educ. 2021;78(3):914–926. doi: 10.1016/j.jsurg.2020.10.002 33109493

[181] Kukulski P , Ward M , Carter K . Ultrasound for volume assessment in patients with shock: effectiveness of an educational intervention for fourth-year medical students. Cureus. 2018;10(1):e2129. doi: 10.7759/cureus.2129 PMC5878096 29610713

[182] Kumar AS . Homeschooling surgery students: Washington state University's longitudinal integrated clerkship transitioned to a hybrid-virtual platform triggered by the COVID-19 pandemic. Am Surg. 2022;88(11):2633–2636. doi: 10.1177/00031348221101518 PMC9561773 36239046

[183] Kumar HB , Khan AM , Arora VK , et al. Fine needle aspiration biopsy: an entrustable professional activity in cytopathology postgraduate training. J Cytol. 2017;34(2):84–89. doi: 10.4103/JOC.JOC_67_16 PMC5398025 28469315

[184] Kwan BYM , Mbanwi A , Cofie N , et al. Creating a competency-based medical education curriculum for Canadian diagnostic radiology residency (Queen's Fundamental Innovations in Residency Education)-Part 1: transition to discipline and foundation of discipline stages. Can Assoc Radiol J. 2021;72(3):372–380. doi: 10.1177/0846537119894723 32126802

[185] Kwan J , Crampton R , Mogensen LL , et al. Bridging the gap: a five stage approach for developing specialty-specific entrustable professional activities. BMC Med Educ. 2016;16:117. doi: 10.1186/s12909-016-0637-x PMC4839106 27097981

[186] Landreville JM , Frank JR , Cheung WJ . Does direct observation happen early in a new competency-based residency program? AEM Educ Train. 2021;5(2):e10591. doi: 10.1002/aet2.10591 PMC8019151 33842816

[187] Landzaat LH , Barnett MD , Buckholz GT , et al. Development of entrustable professional activities for hospice and palliative Medicine fellowship training in the United States. J Pain Symptom Manage. 2017;54(4):609–16 e1. doi: 10.1016/j.jpainsymman.2017.07.003 28729009

[188] Larrabee JG , Agrawal D , Trimm F , et al. Entrustable professional activities: correlation of entrustment assessments of pediatric residents with concurrent subcompetency milestones ratings. J Grad Med Educ. 2020;12(1):66–73. doi: 10.4300/JGME-D-19-00408.1 PMC7012520 32089796

[189] Lee CW , Chen GL , Lee YK . User experience evaluation of the EPAs-Based e-Portfolio system and an analysis of its impact. J Acute Med. 2020;10(3):115–125. 10.6705/j.jacme.202009_10(3).0003 PMC7662102 33209570

[190] Leipzig RM , Sauvigne K , Granville LJ , et al. What is a geriatrician? American geriatrics society and association of directors of geriatric academic programs end-of-training entrustable professional activities for geriatric Medicine. J Am Geriatr Soc. 2014;62(5):924–929. doi: 10.1111/jgs.12825 24749846

[191] Liao N , Leung C , Barbee J , et al. Enter and discuss orders and prescriptions (EPA 4): a curriculum for fourth-year medical students. MedEdPORTAL. 2022;18:11263. doi: 10.15766/mep_2374-8265.11263 PMC9253226 35799675

[192] Liebert CA , Melcer EF , Keehl O , et al. Validity evidence for ENTRUST as an assessment of surgical decision-making for the inguinal hernia entrustable professional activity (EPA). J Surg Educ. 2022;79(6):e202–e12. doi: 10.1016/j.jsurg.2022.07.008 35909070

[193] Lindeman B , Brasel K , Minter RM , et al. A phased approach: the general surgery experience adopting entrustable professional activities in the United States. Acad Med. 2021;96(7S):S9–S13. doi: 10.1097/ACM.0000000000004107 34183596

[194] Lindeman BM , Sacks BC , Lipsett PA . Graduating Students' and surgery program Directors' views of the association of American medical colleges core entrustable professional activities for entering residency: where are the gaps? J Surg Educ. 2015;72(6):e184–92. doi: 10.1016/j.jsurg.2015.07.005 26276302

[195] Linsenmeyer M , Wimsatt L , Speicher M , et al. Status of entrustable professional activities (EPA) implementation at colleges of osteopathic Medicine in the United States and future considerations. J Am Osteopath Assoc. 2020;120(11):749–760. doi: 10.7556/jaoa.2020.129 32946547

[196] Lomis K , Amiel JM , Ryan MS , et al. Implementing an entrustable professional activities framework in undergraduate medical education: early lessons from the AAMC core entrustable professional activities for entering residency pilot. Acad Med. 2017;92(6):765–770. doi: 10.1097/ACM.0000000000001543 28557937

[197] Lum E , van Galen LS , Car J . Competency-based training for entrustment in telehealth consultations. Pediatr Clin North Am. 2020;67(4):735–757. doi: 10.1016/j.pcl.2020.04.013 32650870

[198] Mallow M , Baer H , Moroz A , et al. Entrustable professional activities for residency training in physical Medicine and rehabilitation. Am J Phys Med Rehabil. 2017;96(10):762–764. doi: 10.1097/PHM.0000000000000741 28925925

[199] Martin L , Sibbald M , Brandt Vegas D , et al. The impact of entrustment assessments on feedback and learning: trainee perspectives. Med Educ. 2020;54(4):328–336. doi: 10.1111/medu.14047 31840289

[200] Marty A , Frick S , Bruderer Enzler H , et al. An analysis of core EPAs reveals a gap between curricular expectations and medical school graduates' self-perceived level of competence. BMC Med Educ. 2021;21(1):105. doi: 10.1186/s12909-021-02534-w PMC7885554 33593362

[201] Marty AP , Schmelzer S , Thomasin RA , et al. Agreement between trainees and supervisors on first-year entrustable professional activities for anaesthesia training. Br J Anaesth. 2020;125(1):98–103. doi: 10.1016/j.bja.2020.04.009 32423610

[202] McClintic JA , Snyder CL , Brown KM . Curricular innovation in the surgery clerkship: can assessment methods influence development of critical thinking and clinical skills? J Surg Educ. 2018;75(5):1236–1244. doi: 10.1016/j.jsurg.2018.02.012 29545129

[203] McCloskey CB , Domen RE , Conran RM , et al. Entrustable professional activities for pathology: recommendations from the college of American pathologists graduate medical education committee. Acad Pathol. 2017;4:2374289517714283. doi: 10.1177/2374289517714283 PMC5496684 28725792

[204] McMurray L , Hall AK , Rich J , et al. The nightmares course: a longitudinal, multidisciplinary, simulation-based curriculum to train and assess resident competence in resuscitation. J Grad Med Educ. 2017;9(4):503–508. doi: 10.4300/JGME-D-16-00462.1 PMC5559248 28824766

[205] Meade LB , Suddarth KH , Jones RR , et al. Patients, nurses, and physicians working together to develop a discharge entrustable professional activity assessment tool. Acad Med. 2016;91(10):1388–1391. doi: 10.1097/ACM.0000000000001189 27097051

[206] Meade LB , Todd CY , Walsh MM . Found in transition: applying milestones to three unique discharge curricula. PeerJ. 2015;3:e819. doi: 10.7717/peerj.819 PMC4358664 25780771

[207] Melvin L , Rassos J , Stroud L , et al. Tensions in assessment: the realities of entrustment in internal Medicine. Acad Med. 2020;95(4):609–615. doi: 10.1097/ACM.0000000000002991 31567171

[208] Meyer EG , Harvey E , Durning SJ , et al. Pre-clerkship EPA assessments: a thematic analysis of rater cognition. BMC Med Educ. 2022;22(1):347. doi: 10.1186/s12909-022-03402-x PMC9077896 35524304

[209] Meyer EG , Taylor DR , Uijtdehaage S , et al. EQual rubric evaluation of the association of American medical Colleges' core entrustable professional activities for entering residency. Acad Med. 2020;95(11):1755–1762. doi: 10.1097/ACM.0000000000003504 32433310

[210] Mihaljevic AL , Schmidt J , Mitzkat A , et al. Heidelberger interprofessionelle ausbildungsstation (HIPSTA): a practice- and theory-guided approach to development and implementation of Germany's first interprofessional training ward. GMS J Med Educ. 2018;35(3):Doc33. 10.3205/zma001179 PMC6120150 30186943

[211] Ming D , Zietlow K , Song Y , et al. Discharge summary training curriculum: a novel approach to training medical students how to write effective discharge summaries. Clin Teach. 2019;16(5):507–512. doi: 10.1111/tct.12960 30378265

[212] Mink R , Herman BE , Carraccio C , et al. Agreement of program directors with clinical competency committees for fellow entrustment. J Med Educ Curric Dev. 2020;7:2382120520936613. doi: 10.1177/2382120520936613 PMC7418246 32844115

[213] Mink RB , Myers AL , Turner DA , et al. Competencies, milestones, and a level of supervision scale for entrustable professional activities for scholarship. Acad Med. 2018;93(11):1668–1672. doi: 10.1097/ACM.0000000000002353 29995669

[214] Mink RB , Schwartz A , Herman BE , et al. Validity of level of supervision scales for assessing pediatric fellows on the common pediatric subspecialty entrustable professional activities. Acad Med. 2018;93(2):283–291. doi: 10.1097/ACM.0000000000001820 28700462

[215] Mishra S , Chung A , Rogoza C , et al. Creating a competency-based medical education curriculum for Canadian diagnostic radiology residency (Queen's Fundamental Innovations in Residency Education)-Part 2: core of discipline stage. Can Assoc Radiol J. 2021;72(4):678–685. doi: 10.1177/0846537121993058 33656945

[216] Moeller JJ , Warren JB , Crowe RM , et al. Developing an entrustment process: insights from the AAMC CoreEPA pilot. Med Sci Educ. 2020;30(1):395–401. doi: 10.1007/s40670-020-00918-z PMC8368864 34457683

[217] Moll-Khosrawi P , Ganzhorn A , Zollner C , et al. Development and validation of a postgraduate anaesthesiology core curriculum based on entrustable professional activities: a delphi study. GMS J Med Educ. 2020;37(5):Doc52. 10.3205/zma001345 PMC7499458 32984511

[218] Moore D , Young CJ , Hong J . Implementing entrustable professional activities: the yellow brick road towards competency-based training? ANZ J Surg. 2017;87(12):1001–1005. doi: 10.1111/ans.14120 28768363

[219] Mueller V , Morais M , Lee M , et al. Implementation of entrustable professional activities assessments in a Canadian obstetrics and gynecology residency program: a mixed methods study. Can Med Educ J. 2022;13(5):77–81. doi: 10.36834/cmej.72567 PMC9588190 36310902

[220] Murray KE , Lane JL , Carraccio C , et al. Crossing the gap: using competency-based assessment to determine whether learners are ready for the undergraduate-to-graduate transition. Acad Med. 2019;94(3):338–345. doi: 10.1097/ACM.0000000000002535 30475269

[221] Nayyar B , Yasmeen R , Khan RA . Using language of entrustable professional activities to define learning objectives of radiology clerkship: a modified delphi study. Med Teach. 2019;41(12):1419–1426. doi: 10.1080/0142159X.2019.1645951 31449433

[222] Obeso V , Grbic D , Emery M , et al. Core entrustable professional activities (EPAs) and the transition from medical school to residency: the postgraduate year one resident perspective. Med Sci Educ. 2021;31(6):1813–1822. doi: 10.1007/s40670-021-01370-3 PMC8651854 34956699

[223] O'Connor P , Lydon S , Mongan O , et al. Longitudinal survey of self-reported level of entrustment across the first year of clinical practice. HRB Open Res. 2022;5:9. doi: 10.12688/hrbopenres.13487.1 PMC9005985 35465129

[224] O'Dowd E , Lydon S , O'Connor P , et al. The development of a framework of entrustable professional activities for the intern year in Ireland. BMC Med Educ. 2020;20(1):273. doi: 10.1186/s12909-020-02156-8 PMC7433170 32811490

[225] Marty AP , Braun J , Schick C , et al. A mobile application to facilitate implementation of programmatic assessment in anaesthesia training. Br J Anaesth. 2022;128(6):990–996. doi: 10.1016/j.bja.2022.02.038 35410792

[226] Omoruyi EA , Guardiola A , Barratt MS . Are incoming pediatric interns ready to obtain the essential components of an informed consent for lumbar puncture? Acad Pediatr. 2019;19(4):410–413. doi: 10.1016/j.acap.2018.11.011 30476674

[227] Ong AM , Hum C . Entrust me: embedding entrustable professional activities in a gastroenterology residency program. Dig Dis Sci. 2023;68:352–356. doi: 10.1007/s10620-022-07815-y 36609940

[228] Osborn PM , Dowd TC , Schmitz MR , et al. Establishing an orthopedic program-specific, comprehensive competency-based education program. J Surg Res. 2021;259:399–406. doi: 10.1016/j.jss.2020.09.016 33109403

[229] Pagano MB , Treml A , Stephens LD , et al. Entrustable professional activities for apheresis Medicine education. Transfusion (Paris). 2020;60(10):2432–2440. doi: 10.1111/trf.15983 32757215

[230] Pandya A , Patocka C , Huffman J . Simulation for assessment of entrustable professional activities in an emergency Medicine residency program. CJEM. 2022;24(1):84–87. doi: 10.1007/s43678-021-00209-5 34780048

[231] Parker TA , Guiton G , Jones MD, Jr. Choosing entrustable professional activities for neonatology: a delphi study. J Perinatol. 2017;37(12):1335–1340. doi: 10.1038/jp.2017.144 28933775

[232] Pearlman RE , Pawelczak M , Yacht AC , et al. Program director perceptions of proficiency in the core entrustable professional activities. J Grad Med Educ. 2017;9(5):588–592. doi: 10.4300/JGME-D-16-00864.1 PMC5646915 29075377

[233] Peng CR , Schertzer KA , Caretta-Weyer HA , et al. Assessment of entrustable professional activities using a web-based simulation platform during transition to emergency Medicine residency: mixed methods pilot study. JMIR Med Educ. 2021;7(4):e32356. doi: 10.2196/32356 PMC8663509 34787582

[234] Peters H , Holzhausen Y , Maaz A , et al. Introducing an assessment tool based on a full set of end-of-training EPAs to capture the workplace performance of final-year medical students. BMC Med Educ. 2019;19(1):207. doi: 10.1186/s12909-019-1600-4 PMC6567397 31196063

[235] Pinilla S , Cantisani A , Kloppel S , et al. Introducing a psychiatry clerkship curriculum based on entrustable professional activities: an explorative pilot study. Acad Psychiatry. 2021;45(3):354–359. doi: 10.1007/s40596-021-01417-y PMC8116264 33598804

[236] Pinilla S , Kyrou A , Kloppel S , et al. Workplace-based assessments of entrustable professional activities in a psychiatry core clerkship: an observational study. BMC Med Educ. 2021;21(1):223. doi: 10.1186/s12909-021-02637-4 PMC8059233 33882926

[237] Pinsk M , Karpinski J , Carlisle E . Introduction of competence by design to Canadian nephrology postgraduate training. Can J Kidney Health Dis. 2018;5:2054358118786972. doi: 10.1177/2054358118786972 PMC6050612 30034814

[238] Pitts S , Schwartz A , Carraccio CL , et al. Fellow entrustment for the common pediatric subspecialty entrustable professional activities across subspecialties. Acad Pediatr. 2022;22(6):881–886. doi: 10.1016/j.acap.2021.12.019 34936942

[239] Port N , Weiss A , Maudsley I . Electroconvulsive therapy training: can it be a model of an entrustable professional activity in a competency program? Australas Psychiatry. 2012;20(3):242–245. doi: 10.1177/1039856212447879 22652309

[240] Posel N , Hoover ML , Bergman S , et al. Objective assessment of the entrustable professional activity handover in undergraduate and postgraduate surgical learners. J Surg Educ. 2019;76(5):1258–1266. doi: 10.1016/j.jsurg.2019.03.008 30948340

[241] Post JA , Wittich CM , Thomas KG , et al. Rating the quality of entrustable professional activities: content validation and associations with the clinical context. J Gen Intern Med. 2016;31(5):518–523. doi: 10.1007/s11606-016-3611-8 PMC4835372 26902239

[242] Postmes L , Tammer F , Posthumus I , et al. EPA-based assessment: clinical teachers' challenges when transitioning to a prospective entrustment-supervision scale. Med Teach. 2021;43(4):404–410. doi: 10.1080/0142159X.2020.1853688 33305676

[243] Prudhomme N , O'Brien M , McConnell MM , et al. Relationship between ratings of performance in the simulated and workplace environments among emergency Medicine residents. CJEM. 2020;22(6):811–818. doi: 10.1017/cem.2020.388 32538334

[244] Pruitt Z , Mhaskar R , Kane BG , et al. Development of a health care systems curriculum. Adv Med Educ Pract. 2017;8:745–753. doi: 10.2147/AMEP.S146670 PMC5680896 29138614

[245] Pugh D , Cavalcanti RB , Halman S , et al. Using the entrustable professional activities framework in the assessment of procedural skills. J Grad Med Educ. 2017;9(2):209–214. doi: 10.4300/JGME-D-16-00282.1 PMC5398142 28439355

[246] Quraishi S , Wade W , Black D . Development of a GMC aligned curriculum for internal Medicine including a qualitative study of the acceptability of 'capabilities in practice' as a curriculum model. Future Healthc J. 2019;6(3):196–203. doi: 10.7861/fhj.2018-0016 PMC6798027 31660526

[247] Rennert-May EC , J. M. , Dersch-Mills D , et al. Development of a competency-based medical education curriculum for antimicrobial stewardship. JAMMI. 2019;4(1):6–14. 10.3138/jammi.2018-0020 PMC9603188 36338784

[248] Reyes JA , Greenberg L , Amdur R , et al. Effect of handoff skills training for students during the Medicine clerkship: a quasi-randomized study. Adv Health Sci Educ Theory Pract. 2016;21(1):163–173. doi: 10.1007/s10459-015-9621-1 PMC4749641 26174046

[249] Reynolds TS , Frothingham C , Carreiro JE , et al. Report on 7 Years' experience implementing an undergraduate medical curriculum for osteopathic medical students using entrustable professional activities. J Am Osteopath Assoc. 2020;120(8):529–539. doi: 10.7556/jaoa.2020.087 32717088

[250] Rinaldi I , Findyartini A , Widaty S , et al. Identification and validation of entrustable professional activities in Indonesian internal Medicine residency programs. Acta Med Indones. 2022;54(1):79–96. 35398829

[251] Robson J , Lusman SS , Lee CK , et al. Pediatric gastroenterology, hepatology, and nutrition entrustable professional activities: development of an assessment tool and curricular resources. J Pediatr Gastroenterol Nutr. 2020;71(1):e40–e5. doi: 10.1097/MPG.0000000000002715 32265411

[252] Rodgers V , Tripathi J , Lockeman K , et al. Implementation of a workplace-based assessment system to measure performance of the core entrustable professional activities in the pediatric clerkship. Acad Pediatr. 2021;21(3):564–568. doi: 10.1016/j.acap.2020.09.016 33035730

[253] Rose S , Fix OK , Shah BJ , et al. Entrustable professional activities for gastroenterology fellowship training. Gastroenterology. 2014;147(1):233–242. doi: 10.1053/j.gastro.2014.04.038 24954665

[254] Ryan MS , Richards A , Perera R , et al. Generalizability of the Ottawa surgical competency operating room evaluation (O-SCORE) scale to assess medical student performance on core EPAs in the workplace: findings from one institution. Acad Med. 2021;96(8):1197–1204. doi: 10.1097/ACM.0000000000003921 33464735

[255] Salzman DH , McGaghie WC , Caprio TW , et al. A mastery learning capstone course to teach and assess components of three entrustable professional activities to graduating medical students. Teach Learn Med. 2019;31(2):186–194. doi: 10.1080/10401334.2018.1526689 30596271

[256] Sandhu G , Thompson-Burdine J , Dombrowski J , et al. OpTrust: an effective educational bundle for enhancing faculty-resident intraoperative entrustment. Ann Surg. 2021;273(6):e255–e61. doi: 10.1097/SLA.0000000000003436 33979313

[257] Sauer CG , Robson J , Turmelle YP , et al. North American society for pediatric gastroenterology, hepatology, and nutrition position paper on entrustable professional activities: development of pediatric gastroenterology, hepatology, and nutrition entrustable professional activities. J Pediatr Gastroenterol Nutr. 2020;71(1):136–143. doi: 10.1097/MPG.0000000000002684 32187140

[258] Scheurer JM , Davey C , Pereira AG , et al. Building a shared mental model of competence across the continuum: trainee perceptions of subinternships for residency preparation. J Med Educ Curric Dev. 2021;8:23821205211063350. doi: 10.1177/23821205211063350 PMC8721691 34988291

[259] Schick K , Eissner A , Wijnen-Meijer M , et al. Implementing a logbook on entrustable professional activities in the final year of undergraduate medical education in Germany - a multicentric pilot study. GMS J Med Educ. 2019;36(6):Doc69. 10.3205/zma001277 PMC6905372 31844641

[260] Schmelter V , Marz E , Adolf C , et al. Ward rounds in internal Medicine: validation of an entrustable professional activity (EPA) observation checklist. GMS J Med Educ. 2018;35(2):Doc17. 10.3205/zma001164 PMC6022584 29963607

[261] Schnobrich DJ , Mathews BK , Trappey BE , et al. Entrusting internal Medicine residents to use point of care ultrasound: towards improved assessment and supervision. Med Teach. 2018;40(11):1130–1135. doi: 10.1080/0142159X.2018.1457210 29792102

[262] Schultz K , Griffiths J . Implementing competency-based medical education in a postgraduate family Medicine residency training program: a stepwise approach, facilitating factors, and processes or steps that would have been helpful. Acad Med. 2016;91(5):685–689. doi: 10.1097/ACM.0000000000001066 26717504

[263] Schultz K , Griffiths J , Lacasse M . The application of entrustable professional activities to inform competency decisions in a family Medicine residency program. Acad Med. 2015;90(7):888–897. doi: 10.1097/ACM.0000000000000671 25719674

[264] Schumacher DJ , Martini A , Kinnear B , et al. Facilitators and inhibitors to assessing entrustable professional activities in pediatric residency. Acad Pediatr. 2021;21(4):735–741. doi: 10.1016/j.acap.2020.11.013 33221495

[265] Schumacher DJ , Schwartz A , Zenel Jr. JA , et al. Narrative performance level assignments at initial entrustment and graduation: integrating EPAs and milestones to improve learner assessment. Acad Med. 2020;95(11):1736–1744. doi: 10.1097/ACM.0000000000003300 32195689

[266] Schumacher DJ , West DC , Schwartz A , et al. Longitudinal assessment of resident performance using entrustable professional activities. JAMA Netw Open. 2020;3(1):e1919316. doi: 10.1001/jamanetworkopen.2019.19316 PMC6991321 31940042

[267] Schwartz A , Balmer DF , Borman-Shoap E , et al. Shared mental models among clinical competency committees in the context of time-variable, competency-based advancement to residency. Acad Med. 2020;95(11S Association of American Medical Colleges Learn Serve Lead: Proceedings of the 59th Annual Research in Medical Education Presentations):S95–S102. 10.1097/ACM.0000000000003638 32769469

[268] Schwartz A , Borman-Shoap E , Carraccio C , et al. Learner levels of supervision across the continuum of pediatrics training. Acad Med. 2021;96(7S):S42–S9. 10.1097/ACM.0000000000004095 34183601

[269] Sevey N , Barratt M , Omoruyi E . Can incoming United States pediatric interns be entrusted with the essential communication skills of informed consent? J Educ Eval Health Prof. 2020;17:18. doi: 10.3352/jeehp.2020.17.18 PMC7403531 32602671

[270] Sharma P , Tanveer N , Goyal A . A search for entrustable professional activities for the 1(st) year pathology postgraduate trainees. J Lab Physicians. 2018;10(1):26–30. doi: 10.4103/JLP.JLP_51_17 PMC5784288 29403200

[271] Shaughnessy AF , Chang KT , Sparks J , et al. Assessing and documenting the cognitive performance of family Medicine residents practicing outpatient Medicine. J Grad Med Educ. 2014;6(3):526–531. doi: 10.4300/JGME-D-13-00341.1 PMC4535219 26279780

[272] Shaughnessy AF , Sparks J , Cohen-Osher M , et al. Entrustable professional activities in family Medicine. J Grad Med Educ. 2013;5(1):112–118. doi: 10.4300/JGME-D-12-00034.1 PMC3613294 24404237

[273] Shaw RD , Lamb CR , Carroll MC , et al. Implementation of a standardized sub-intern curriculum improves confidence for those entering a surgical residency. J Surg Educ. 2022;79(6):1402–1412. doi: 10.1016/j.jsurg.2022.07.002 35868970

[274] Sherbino J , Bandiera G , Doyle K , et al. The competency-based medical education evolution of Canadian emergency Medicine specialist training. CJEM. 2020;22(1):95–102. doi: 10.1017/cem.2019.417 31965965

[275] Sheth M , Woods RW , Slanetz PJ , et al. Development of breast imaging specific entrustable professional activities using a double delphi technique. Acad Radiol. 2019;26(5):591–596. doi: 10.1016/j.acra.2018.10.023 31047102

[276] Shrestha S , Shrestha A , Shah JN , et al. Competency based post graduate residency program at patan academy of health sciences, Nepal. J Nepal Health Res Counc. 2021;19(1):189–195. doi: 10.33314/jnhrc.v19i1.3263 33934158

[277] Shrivastava SR , Chacko TV , Bhandary S , et al. Development, validation and use of appropriate assessment tools for certification of entrustable professional activities in community Medicine to produce a competent postgraduate: a pilot study. Indian J Public Health. 2019;63(4):277–281. doi: 10.4103/ijph.IJPH_45_19 32189644

[278] Shumway NM , Dacus JJ , Lathrop KI , et al. Use of milestones and development of entrustable professional activities in 2 Hematology/Oncology training programs. J Grad Med Educ. 2015;7(1):101–104. doi: 10.4300/JGME-D-14-00283.1 PMC4507897 26217433

[279] Silva C , Carozza Colombini MN , Luiz Passarela M , et al. [On the need for supervision of the entrants to medical residences]. Medicina (B Aires). 2019;79(5):384–390. 31671388

[280] Sinclair T , Bell BI , Perez K , et al. Rapid mobilization of medical student volunteers to administer vaccines during the COVID-19 pandemic. J Med Educ Curric Dev. 2022;9:23821205211073092. doi: 10.1177/23821205211073092 PMC8755923 35036568

[281] Smit MP , de Hoog M , Brackel HJL , et al. A national process to enhance the validity of entrustment decisions for Dutch pediatric residents. J Grad Med Educ. 2019;11(4 Suppl):158–164. doi: 10.4300/JGME-D-18-01006 PMC6697299 31428274

[282] Sohrmann M , Berendonk C , Nendaz M , et al. Nationwide introduction of a new competency framework for undergraduate medical curricula: a collaborative approach. Swiss Med Wkly. 2020;150:w20201. doi: 10.57187/smw.2020.20201 32294223

[283] Sood L , Emke AR , Castillo JM , et al. Entrustable professional activities: a faculty development workshop for adding EPAs into your medical Trainee's assessment portfolio. MedEdPORTAL. 2017;13:10528. doi: 10.15766/mep_2374-8265.10528 PMC6342153 30800730

[284] Soran C , Laponis R , Summerville S , et al. Identifying entrustable professional activities for internal Medicine residents in ambulatory continuity practice. J Gen Intern Med. 2020;35(6):1917–1919. doi: 10.1007/s11606-019-05430-8 PMC7280367 31637647

[285] Soukoulis V , Gusic ME . How a year of clinical experience affects Students' perspectives about the supervision they need to perform entrustable professional activities. Med Sci Educ. 2019;29(4):915–918. doi: 10.1007/s40670-019-00788-0 PMC8368485 34457565

[286] Soukoulis V , Martindale J , Bray MJ , et al. The use of EPA assessments in decision-making: do supervision ratings correlate with other measures of clinical performance? Med Teach. 2021;43(11):1323–1329. doi: 10.1080/0142159X.2021.1947480 34242113

[287] Stahl CC , Collins E , Jung SA , et al. Implementation of entrustable professional activities into a general surgery residency. J Surg Educ. 2020;77(4):739–748. doi: 10.1016/j.jsurg.2020.01.012 PMC7305986 32044326

[288] Stahl CC , Jung SA , Rosser AA , et al. Entrustable professional activities in general surgery: trends in resident self-assessment. J Surg Educ. 2020;77(6):1562–1567. doi: 10.1016/j.jsurg.2020.05.005 32540120

[289] Stave EA , Greenberg L , Hamburger E , et al. An educational intervention to facilitate appropriate subspecialty referrals: a study assessing resident communication skills. BMC Med Educ. 2022;22(1):533. doi: 10.1186/s12909-022-03592-4 PMC9270829 35804336

[290] Steiman J , Sullivan SA , Scarborough J , et al. Measuring competence in surgical training through assessment of surgical entrustable professional activities. J Surg Educ. 2018;75(6):1452–1462. doi: 10.1016/j.jsurg.2018.05.004 30097351

[291] Steinemann S , Gardner A , Aulet T , et al. American college of surgeons/Association for surgical education medical student simulation-based surgical skills curriculum: alignment with entrustable professional activities. Am J Surg. 2019;217(2):198–204. doi: 10.1016/j.amjsurg.2018.10.012 30497660

[292] Stephan A , Cheung G , van der Vleuten C . Entrustable professional activities and learning: the postgraduate trainee perspective. Acad Psychiatry. 2022;47:134–142. doi: 10.1007/s40596-022-01712-2 PMC10060374 36224504

[293] Stoneham EJ , Witt L , Paterson QS , et al. The development of entrustable professional activities reference cards to support the implementation of competence by design in emergency Medicine. CJEM. 2019;21(6):803–806. doi: 10.1017/cem.2019.395 31771686

[294] Strowd RE , McBride A , Goforth J , et al. Educational priorities of students in the entrustable professional activity era. Clin Teach. 2018;15(4):319–324. doi: 10.1111/tct.12688 28857486

[295] Stucke RS , Sorensen M , Rosser A , et al. The surgical consult entrustable professional activity (EPA): defining competence as a basis for evaluation. Am J Surg. 2020;219(2):253–257. doi: 10.1016/j.amjsurg.2018.12.056 30616922

[296] Taylor DR , Park YS , Egan R , et al. EQual, a novel rubric to evaluate entrustable professional activities for quality and structure. Acad Med. 2017;92(11S Association of American Medical Colleges Learn Serve Lead: Proceedings of the 56th Annual Research in Medical Education Sessions):S110–S7. 10.1097/ACM.0000000000001908 29065031

[297] Taylor DR , Park YS , Smith CA , et al. Creating entrustable professional activities to assess internal Medicine residents in training: a mixed-methods approach. Ann Intern Med. 2018;168(10):724–729. doi: 10.7326/M17-1680 29710333

[298] Ten Cate O , Graafmans L , Posthumus I , et al. The EPA-based Utrecht undergraduate clinical curriculum: development and implementation. Med Teach. 2018;40(5):506–513. doi: 10.1080/0142159X.2018.1435856 29468913

[299] Thoma B , Hall AK , Clark K , et al. Evaluation of a national competency-based assessment system in emergency Medicine: a CanDREAM study. J Grad Med Educ. 2020;12(4):425–434. doi: 10.4300/JGME-D-19-00803.1 PMC7450748 32879682

[300] Thompson LR , Leung CG , Green B , et al. Development of an assessment for entrustable professional activity (EPA) 10: emergent patient management. West J Emerg Med. 2017;18(1):35–42. doi: 10.5811/westjem.2016.10.31479 PMC5226760 28116006

[301] Tomiak A , Braund H , Egan R , et al. Exploring how the new entrustable professional activity assessment tools affect the quality of feedback given to medical oncology residents. J Cancer Educ. 2020;35(1):165–177. doi: 10.1007/s13187-018-1456-z 30604387

[302] Tomiak A , Linford G , McDonald M , et al. Implementation of competency-based medical education in a Canadian medical oncology training program: a first year retrospective review. J Cancer Educ. 2022;37(3):852–856. doi: 10.1007/s13187-020-01895-y 33108804

[303] Touchie C , De Champlain A , Pugh D , et al. Supervising incoming first-year residents: faculty expectations versus residents' experiences. Med Educ. 2014;48(9):921–929. doi: 10.1111/medu.12503 25113118

[304] Traba C , Holland B , Laboy MC , et al. A multi-modal remote clinical skills mini-course utilizing a teaching TeleOSCE. Med Sci Educ. 2021;31(2):503–509. doi: 10.1007/s40670-020-01201-x PMC7787878 33432278

[305] Turner DA , Schwartz A , Carraccio C , et al. Continued supervision for the common pediatric subspecialty entrustable professional activities May be needed following fellowship graduation. Acad Med. 2021;96(7S):S22–S8. 10.1097/ACM.0000000000004091 34183598

[306] Unaka NI , Winn A , Spinks-Franklin A , et al. An entrustable professional activity addressing racism and pediatric health inequities. Pediatrics. 2022;149(2). doi: 10.1542/peds.2021-054604 PMC9647957 35001103

[307] Valentine N , Wignes J , Benson J , et al. Entrustable professional activities for workplace assessment of general practice trainees. Med J Aust. 2019;210(8):354–359. doi: 10.5694/mja2.50130 30977150

[308] van Bockel EAP , Walstock PA , van Mook W , et al. Entrustable professional activities (EPAs) for postgraduate competency based intensive care Medicine training in the Netherlands: the next step towards excellence in intensive care Medicine training. J Crit Care. 2019;54:261–267. doi: 10.1016/j.jcrc.2019.09.012 31733630

[309] van Bruggen L , van Dijk EE , van der Schaaf M , et al. Developing entrustable professional activities for university teachers in the health professions. Med Teach. 2022;44(4):425–432. doi: 10.1080/0142159X.2021.1998402 34767494

[310] van Dam M , Ramani S , Ten Cate O . An EPA for better bedside teaching. Clin Teach. 2021;18(4):398–403. doi: 10.1111/tct.13346 PMC8451813 33763984

[311] van Lieshout JH , Malzkorn B , Steiger HJ , et al. Defining activities in neurovascular microsurgery training: entrustable professional activities for vascular neurosurgery. Acta Neurochir (Wien). 2023;165(1):27–37. doi: 10.1007/s00701-022-05372-x PMC9840588 36271161

[312] van Loon KA , Bonnie LHA , van Dijk N , et al. Benefits of EPAs at risk? The influence of the workplace environment on the uptake of EPAs in EPA-based curricula. Perspect Med Educ. 2021;10(4):200–206. doi: 10.1007/S40037-021-00658-9 PMC8368760 33788161

[313] van Loon KA , Teunissen PW , Driessen EW , et al. The role of generic competencies in the entrustment of professional activities: a nationwide competency-based curriculum assessed. J Grad Med Educ. 2016;8(4):546–552. doi: 10.4300/JGME-D-15-00321.1 PMC5058587 27777665

[314] Villaizan Perez C , Aparicio Rodrigo M , Kovacs A , et al. Entrustable professional activities for the training of postgraduate primary care pediatricians. Acad Med. 2022;98:468–472. doi: 10.1097/ACM.0000000000005132 36584098

[315] Violato C , Cullen MJ , Englander R , et al. Validity evidence for assessing entrustable professional activities during undergraduate medical education. Acad Med. 2021;96(7S):S70–S5. 10.1097/ACM.0000000000004090 34183605

[316] Wagner JP , Lewis CE , Tillou A , et al. Use of entrustable professional activities in the assessment of surgical resident competency. JAMA Surg. 2018;153(4):335–343. doi: 10.1001/jamasurg.2017.4547 PMC5933400 29141086

[317] Wang PZT , Chan E , Forster A , et al. Perceptions on competence by design in urology. Can Urol Assoc J. 2018;13:E183–E9. doi: 10.5489/cuaj.5610 PMC6667332 30472984

[318] Warm EJ , Mathis BR , Held JD , et al. Entrustment and mapping of observable practice activities for resident assessment. J Gen Intern Med. 2014;29(8):1177–1182. doi: 10.1007/s11606-014-2801-5 PMC4099463 24557518

[319] Weiss A , Ozdoba A , Carroll V , et al. Entrustable professional activities: enhancing meaningful use of evaluations and milestones in a psychiatry residency program. Acad Psychiatry. 2016;40(5):850–854. doi: 10.1007/s40596-016-0530-2 26976398

[320] Weiss P , Schwartz A , Carraccio C , et al. Minimum supervision levels required by program directors for pediatric pulmonary fellow graduation. ATS Sch. 2021;2(3):360–369. doi: 10.34197/ats-scholar.2021-0013OC PMC8519336 34667986

[321] Weiss PG , Schwartz A , Carraccio CL , et al. Achieving entrustable professional activities during fellowship. Pediatrics. 2021;148(5). doi: 10.1542/peds.2021-050196 34667096

[322] Weissenbacher A , Bolz R , Stehr SN , et al. Development and consensus of entrustable professional activities for final-year medical students in anaesthesiology. BMC Anesthesiol. 2022;22(1):128. doi: 10.1186/s12871-022-01668-8 PMC9052481 35488205

[323] White K , Qualtieri J , Courville EL , et al. Entrustable professional activities in hematopathology pathology fellowship training: consensus design and proposal. Acad Pathol. 2021;8:2374289521990823. doi: 10.1177/2374289521990823 PMC7894592 33644302

[324] Wijeratne DT , Srivastava S , Chan B , et al. Sustainability of physical exam skills in a resident-led curriculum in a large internal Medicine program with competency based medical education. Can Med Educ J. 2018;9(4):e78–e92. doi: 10.36834/cmej.43273 PMC6260518 30498546

[325] Wijnen-Meijer M , Brandhuber T , Schneider A , et al. Implementing kolb s experiential learning cycle by linking real experience, case-based discussion and simulation. J Med Educ Curric Dev. 2022;9:23821205221091511. doi: 10.1177/23821205221091511 PMC9112303 35592131

[326] Wijnen-Meijer M , Ten Cate O , van der Schaaf M , et al. Vertically integrated medical education and the readiness for practice of graduates. BMC Med Educ. 2015;15:229. doi: 10.1186/s12909-015-0514-z PMC4687104 26689282

[327] Winn AS , Marcus CH , Sectish TC , et al. A comparison of resident self-perception and pediatric hospitalist perceptions of the supervisory needs of new interns. Hosp Pediatr. 2018;8(4):214–219. doi: 10.1542/hpeds.2017-0212 29535206

[328] Wisman-Zwarter N , van der Schaaf M , Ten Cate O , et al. Transforming the learning outcomes of anaesthesiology training into entrustable professional activities: a delphi study. Eur J Anaesthesiol. 2016;33(8):559–567. doi: 10.1097/EJA.0000000000000474 27270884

[329] Wolfel T , Beltermann E , Lottspeich C , et al. Medical ward round competence in internal Medicine - an interview study towards an interprofessional development of an entrustable professional activity (EPA). BMC Med Educ. 2016;16:174. doi: 10.1186/s12909-016-0697-y PMC4940908 27401103

[330] Woods R , Singh S , Thoma B , et al. Validity evidence for the quality of assessment for learning score: a quality metric for supervisor comments in competency based medical education. Can Med Educ J. 2022;13(6):19–35. doi: 10.36834/cmej.74860 PMC9684040 36440075

[331] Woodworth GE , Marty AP , Tanaka PP , et al. Development and pilot testing of entrustable professional activities for US anesthesiology residency training. Anesth Analg. 2021;132(6):1579–1591. doi: 10.1213/ANE.0000000000005434 33661789

[332] Wurster Ovalle V , Martini A , Tanguay S , et al. Implementing the behavioral and mental health entrustable professional activity: insights for a path forward. Acad Pediatr. 2021;21(1):178–184. doi: 10.1016/j.acap.2020.07.002 32645533

[333] Yilmaz Y , Carey R , Chan TM , et al. Developing a dashboard for faculty development in competency-based training programs: a design-based research project. Can Med Educ J. 2021;12(4):48–64. doi: 10.36834/cmej.72067 PMC8463237 34567305

[334] Yilmaz Y , Carey R , Chan TM , et al. Developing a dashboard for program evaluation in competency-based training programs: a design-based research project. Can Med Educ J. 2022;13(5):14–27. doi: 10.36834/cmej.73554 PMC9588183 36310899

[335] Younas A , Khan RA , Yasmin R . Entrustment in physician-patient communication: a modified delphi study using the EPA approach. BMC Med Educ. 2021;21(1):497. doi: 10.1186/s12909-021-02931-1 PMC8454034 34544394

[336] Young JQ , Hasser C , Hung EK , et al. Developing end-of-training entrustable professional activities for psychiatry: results and methodological lessons. Acad Med. 2018;93(7):1048–1054. doi: 10.1097/ACM.0000000000002058 29166349

[337] Young JQ , McClure M . Fast, easy, and good: assessing entrustable professional activities in psychiatry residents with a mobile app. Acad Med. 2020;95(10):1546–1549. doi: 10.1097/ACM.0000000000003390 32271227

[338] Young JQ , Sugarman R , Schwartz J , et al. A mobile app to capture EPA assessment data: utilizing the consolidated framework for implementation research to identify enablers and barriers to engagement. Perspect Med Educ. 2020;9(4):210–219. doi: 10.1007/S40037-020-00587-Z PMC7459074 32504446

[339] Yu HY , Everett EN , Baecher-Lind L , et al. Feasibility of a regional approach to an obstetrics and gynecology transition to residency course. R I Med J (2013). 2019;102(7):51–55. 31480822

[340] Yuan CM , Prince LK , Oliver JD , et al. Implementation of nephrology subspecialty curricular milestones. Am J Kidney Dis. 2015;66(1):15–22. doi: 10.1053/j.ajkd.2015.01.020 25773484

[341] Yuan CM , Prince LK , Zwettler AJ , et al. Assessing achievement in nephrology training: using clinic chart audits to quantitatively screen competency. Am J Kidney Dis. 2014;64(5):737–743. doi: 10.1053/j.ajkd.2014.06.027 25156904

[342] Yun Z , Jing L , Junfei C , et al. Entrustable professional activities for Chinese standardized residency training in pediatric intensive care Medicine. Front Pediatr. 2022;10:919481. doi: 10.3389/fped.2022.919481 PMC9289143 35859946

[343] Zackoff MW , Young D , Sahay RD , et al. Establishing objective measures of clinical competence in undergraduate medical education through immersive virtual reality. Acad Pediatr. 2021;21(3):575–579. doi: 10.1016/j.acap.2020.10.010 PMC7572369 33091608

[344] Gummesson C , Alm S , Cederborg A , et al. Entrustable professional activities (EPAs) for undergraduate medical education - development and exploration of social validity. BMC Med Educ. 2023;23(1):635. doi: 10.1186/s12909-023-04621-6 PMC10478490 37667366

[345] Gomez-Garibello C , Wagner M , Seymour N , et al. The entrustable professional activities of laparoscopic surgery: moving toward an integrated training model. Surg Endosc. 2023;37(7):5335–5339. doi: 10.1007/s00464-023-10022-z PMC10054191 36988666

[346] Cadieux M , McKinley SK , Odewade N , et al. Neurosurgeons' perspectives on vascular entrustable professional activities. Can J Neurol Sci. 2023;50(2):287–289. doi: 10.1017/cjn.2021.528 35190007

[347] Hanna K , Gupta S , Hurst R , et al. Specialty-specific entrustable professional activities: a bridge to internship. Cureus. 2023;15(2):e35547. doi: 10.7759/cureus.35547 PMC10057663 37007399

[348] Paisant A , Skehan S , Colombie M , et al. Development and validation of core entrustable professional activities for abdominal radiology. Insights Imaging. 2023;14(1):142. doi: 10.1186/s13244-023-01482-x PMC10465426 37644337

[349] Encandela JA , Shaull L , Jayas A , et al. Entrustable professional activities as a training and assessment framework in undergraduate medical education: a case study of a multi-institutional pilot. Med Educ Online. 2023;28(1):2175405. doi: 10.1080/10872981.2023.2175405 PMC9937016 36794397

[350] Yan F , Yang X , Zhang L , et al. Establishing entrustable professional activities for psychiatry residents in China. BMC Med Educ. 2023;23(1):623. doi: 10.1186/s12909-023-04583-9 PMC10474625 37658351

[351] Ganzhorn A , Schulte-Uentrop L , Kullmei J , et al. National consensus on entrustable professional activities for competency-based training in anaesthesiology. PLoS One. 2023;18(7):e0288197. doi: 10.1371/journal.pone.0288197 PMC10335696 37432949

[352] Eymann A , Silva C , Soto Perez AR , et al. Autonomy in entrustable professional activities after the COVID-19 pandemic: the perspectives of residents and teachers. Arch Argent Pediatr. 2023;121(5):e202302996. doi: 10.5546/aap.2023-02996.eng 37594490

[353] Roth LT , Cooper MB , Lurie B , et al. Developing an entrustable professional activity to improve the care of LGBTQ+ youth. Acad Pediatr. 2023;23(4):697–702. doi: 10.1016/j.acap.2022.09.006 36122833

[354] Rafiq A , Sethi A . Entrustable professional activities for bedside clinical teachers. BMC Med Educ. 2024;24(1):887. doi: 10.1186/s12909-024-05876-3 PMC11331656 39155387

[355] Benstead K , Brandl A , Brouwers T , et al. An inter-specialty cancer training programme curriculum for Europe. Eur J Surg Oncol. 2023;49(9):106989. doi: 10.1016/j.ejso.2023.106989 37556988

[356] Bryant BH , Anderson SR , Brissette M , et al. National pilot of entrustable professional activities in pathology residency training. Acad Pathol. 2024;11(2):100110. doi: 10.1016/j.acpath.2024.100110 PMC10978478 38560425

[357] Brian R , Rodriguez N , Zhou CJ , et al. Doing well": intraoperative entrustable professional activity assessments provided limited technical feedback. Surg Open Sci. 2024;18:93–97. doi: 10.1016/j.sopen.2024.02.008 PMC10907196 38435485

[358] Pinilla S , Bauer W , Breckwoldt J , et al. Introducing entrustable professional activities for postgraduate medical training in Switzerland. GMS J Med Educ. 2024;41(5):Doc60. 10.3205/zma001715 PMC11656180 39711863

[359] Mink RB , Carraccio CL , Herman BE , et al. Relationship between epa level of supervision with their associated subcompetency milestone levels in pediatric fellow assessment. BMC Med Educ. 2023;23(1):720. doi: 10.1186/s12909-023-04689-0 PMC10548580 37789289

[360] Mashayekhi J , Mafinejad MK . Mapping the core competencies and entrustable professional activities of medical ethics for faculty members. BMC Med Educ. 2023;23(1):409. doi: 10.1186/s12909-023-04305-1 PMC10242784 37277754

[361] European society of R. Resident education in radiology in Europe including entrustable professional activities: results of an ESR survey. Insights Imaging. 2023;14(1):139. doi: 10.1186/s13244-023-01489-4 PMC10444922 37606861

[362] Violato C , Englander R , Dale E , et al. Implementing core entrustable professional activities in undergraduate medical education: a psychometric study. Acad Med. 2024;100:585–591. doi: 10.1097/ACM.0000000000005907 39485120

[363] de Laat JM , van der Horst-Schrivers ANA , Appelman-Dijkstra NM , et al. Assessment of entrustable professional activities among Dutch endocrine supervisors. J CME. 2024;13(1):2360137. doi: 10.1080/28338073.2024.2360137 PMC11146265 38831939

[364] Pillai P , Maamouri A , Temte J . Identifying community-based entrustable professional activities for medical students through a modified delphi process. Fam Med. 2024;56(8):501–504. doi: 10.22454/FamMed.2024.818912 PMC11412285 39012287

[365] Costa ML , Rego MAS , Rodrigues FC , et al. Validation of entrustable professional activities for use in neonatal care residency programs. J Pediatr (Rio J. 2024;100(6):627–632. doi: 10.1016/j.jped.2024.05.003 PMC11662742 38955326

[366] Montgomery KB , Mellinger JD , Jones A , et al. Validity of entrustable professional activities in a national sample of general surgery residency programs. J Am Coll Surg. 2024;238(4):376–384. doi: 10.1097/XCS.0000000000000967 PMC10942265 38224150

[367] Bremer AE , Ruczynski LIA , Bot P , et al. A qualitative study on how entrustable professional activities support medical students in their transitions across clerkships. Perspect Med Educ. 2023;12(1):187–197. doi: 10.5334/pme.825 PMC10237239 37274808

[368] Andreou V , Peters S , Eggermont J , et al. Evaluating feedback comments in entrustable professional activities: a cross-sectional study. J Med Educ Curric Dev. 2024;11:23821205241275810. doi: 10.1177/23821205241275810 PMC11437546 39346122

[369] Dhami N , Hamza DM , Daniels VJ . Motivations for entrustable professional activity assessment: gaps between curriculum theory and resident reality. J Grad Med Educ. 2024;16(2):166–174. doi: 10.4300/JGME-D-23-00470.1 PMC11234313 38993299

[370] Andreou V , Peters S , Eggermont J , et al. Co-designing entrustable professional activities in general Practitioner's training: a participatory research study. BMC Med Educ. 2024;24(1):549. doi: 10.1186/s12909-024-05530-y PMC11100052 38760773

[371] Aschwanden FJ , Afferi L , Kandler L , et al. Mobile-based assessment of entrustable professional activities in urology training: implementation and outcomes. Eur Urol Open Sci. 2025;73:71–76. doi: 10.1016/j.euros.2025.01.013 PMC11889345 40060303

[372] Gama A , Felicelli C , Agarwal I , et al. Entrustable professional activities (EPAs) in surgical pathology: implementation experience and longitudinal observations of resident development. Acad Pathol. 2024;11(4):100150. doi: 10.1016/j.acpath.2024.100150 PMC11541477 39512707

[373] Singh S , Cheung WJ , Dewhirst S , et al. The influence of clinical coaching teams on quality of entrustable professional activity assessments. AEM Educ Train. 2023;7(3):e10879. doi: 10.1002/aet2.10879 PMC10290210 37361186

[374] Mash R , Jenkins L , Naidoo M . Development of entrustable professional activities for family Medicine in South Africa. Afr J Prim Health Care Fam Med. 2024;16(1):e1–e5. doi: 10.4102/PHCFM.v16i1.4483 PMC11019106 38572863

[375] Sheikh N , Mehta J , Shah R , et al. Feedback that lands: exploring how residents receive and judge feedback during entrustable professional activities. Perspect Med Educ. 2023;12(1):427–437. doi: 10.5334/pme.1020 PMC10588547 37868076

[376] Madrazo L , Correa JDC , Puka N , et al. SL. Evaluating the quality of written feedback within entrustable professional activities in an internal Medicine cohort. J Grad Med Educ. 2023;15(1):74–80. doi: 10.4300/JGME-D-22-00222.1 PMC9934834 36817541

[377] Matava CT , Alam F , Kealey A , et al. The influence of resident and faculty gender on assessments in anesthesia competency-based medical education. Can J Anaesth. 2023;70(6):978–987. doi: 10.1007/s12630-023-02454-x PMC10171726 37165126

[378] Villa S , Caretta-Weyer H , Yarris LM , et al. Development of entrustable professional activities for emergency Medicine medical education fellowships: a modified delphi study. AEM Educ Train. 2024;8(1):e10944. doi: 10.1002/aet2.10944 PMC10950010 38504805

[379] Paterson QS , Alrimawi H , Sample S , et al. Examining enablers and barriers to entrustable professional activity acquisition using the theoretical domains framework: a qualitative framework analysis study. AEM Educ Train. 2023;7(2):e10849. doi: 10.1002/aet2.10849 PMC10041073 36994315

[380] Fisk DCP , Clendenning BG , St John PD , et al. Multi-stakeholder validation of an entrustable professional activities framework for Canadian geriatrics residency programs. Can Geriatr J. 2024;27(3):281–289. doi: 10.5770/cgj.27.728 PMC11346625 39234279

[381] Guo FC , Chen YT , Hsu WC , et al. EMYWAY workplace-based entrustable professional activities assessments in otolaryngology residency training: a nationwide experience. Otolaryngol Head Neck Surg. 2025;172(4):1242–1253. doi: 10.1002/ohn.1104 PMC11947863 39739526

[382] Stone SN , Rydberg L . Creating and confirming observable professional activities (OPAs): a brief report on the practical approach for OPA design for resident education. J Spinal Cord Med. 2023;46(5):865–869. doi: 10.1080/10790268.2023.2191100 PMC10446771 36972220

[383] Ahn E , LaDonna KA , Landreville JM , et al. Only as strong as the weakest link: resident perspectives on entrustable professional activities and their impact on learning. J Grad Med Educ. 2023;15(6):676–684. doi: 10.4300/JGME-D-23-00204.1 PMC10686661 38045932

[384] Ha EL , Glaeser AM , Wilhalme H , et al. Assessing readiness: the impact of an experiential learning entrustable professional activity-based residency preparatory course. Med Educ Online. 2024;29(1):2352217. doi: 10.1080/10872981.2024.2352217 PMC11104695 38758979

[385] Jones Jr. MD , Guiton G , Yost CC , et al. Structured debriefing to assess performance of entrustable professional activities. J Grad Med Educ. 2024;16(5):607–610. doi: 10.4300/JGME-D-24-00247.1 PMC11475426 39416407

[386] Felicelli C , Gama A , Chornenkyy Y , et al. From principles to practice: implementation of entrustable professional activities (EPAs) for surgical pathology residency education in a large academic hospital. Acad Pathol. 2023;10(4):100097. doi: 10.1016/j.acpath.2023.100097 PMC10679497 38025045

[387] Ten Cate O , Nel D , Hennus MP , et al. For which entrustable professional activities must medical students be prepared if unsupervised patient care without further training is an expectation? An international global south study. BMJ Glob Health. 2024;9(10):e016090. doi: 10.1136/bmjgh-2024-016090 PMC11481114 39414331

[388] Buyck M , Desaulniers P , Chenier C , et al. Experiential faculty development to increase the number of entrustable professional activity assessments. Clin Teach. 2025;22(1):e70006. doi: 10.1111/tct.70006 PMC11669488 39721718

[389] Chang YC , Khwepeya M , Nkambule NS , et al. Emergency residents' self-perceived readiness for practice: the association of milestones, entrustable professional activities, and professional identities-a multi-institutional survey. Front Med (Lausanne). 2023;10:1032516. doi: 10.3389/fmed.2023.1032516 PMC10213224 37250629

[390] Cifra N , Pitts S , Mink R , et al. Analysis of fellowship program director opinions of entrustable professional activities in adolescent Medicine fellowship. Int J Adolesc Med Health. 2024;36(3):237–242. doi: 10.1515/ijamh-2023-0154 38522004

[391] Dougherty R , Fornari A , Farina G , et al. Medical error disclosure: an entrustable professional activity during an objective standardized clinical examination for clerkship students. MedEdPORTAL. 2024;20:11382. doi: 10.15766/mep_2374-8265.11382 PMC10876916 38380273

[392] Berger S , Stalmeijer RE , Marty AP , et al. Exploring the impact of entrustable professional activities on feedback culture: a qualitative study of anesthesiology residents and attendings. Acad Med. 2023;98(7):836–843. doi: 10.1097/ACM.0000000000005188 36812061

[393] Lin DT , Melcer EF , Keehl O , et al. ENTRUST: a serious game-based virtual patient platform to assess entrustable professional activities in graduate medical education. J Grad Med Educ. 2023;15(2):228–236. doi: 10.4300/JGME-D-22-00518.1 PMC10150817 37139206

[394] van Enk A , MacDonald G , Hatala R , et al. Not in the file: how competency committees work with undocumented contributions. Med Educ. 2024;58(11):1333–1342. doi: 10.1111/medu.15457 38899368

[395] Li S , Qi X , Li H , et al. Exploration of validity evidence for core residency entrustable professional activities in Chinese pediatric residency. Front Med (Lausanne). 2023;10:1301356. doi: 10.3389/fmed.2023.1301356 PMC10801054 38259855

[396] Lerch SP , Pinilla S , Nendaz M , et al. Trainee doctors' preparedness for clinical work in geriatric psychiatry: a survey on 18 preliminary entrustable professional activities. Int J Geriatr Psychiatry. 2023;38(6):e5954. doi: 10.1002/gps.5954 37344928

[397] Xu B , Zhang J , Ye L , et al. Constructing an evaluation and assessment system for urology specialists based on entrustable professional activities. Arch Esp Urol. 2024;77(9):999–1006. doi: 10.56434/j.arch.esp.urol.20247709.142 39632522

[398] Wang L , Zhao Y , Wang P , et al. Application of clinical thinking training system based on entrustable professional activities in emergency teaching. BMC Med Educ. 2024;24(1):1294. doi: 10.1186/s12909-024-06302-4 PMC11559239 39533290

[399] Rogoza C , Fasih S , Kwan BYM . Implementing a competency based medical education curriculum in diagnostic radiology: challenges and pearls of wisdom. Curr Probl Diagn Radiol. 2025;54:543–545. doi: 10.1067/j.cpradiol.2025.01.012 39904679

[400] Karpinski J , Stewart J , Oswald A , et al. Competency-based medical education at scale: a road map for transforming national systems of postgraduate medical education. Perspect Med Educ. 2024;13(1):24–32. doi: 10.5334/pme.957 PMC10870941 38371306

[401] Suneja M , Thoma KD , Franklin E , et al. Pivoting during a pandemic: reimagining intern orientation EPA-Based OSCE. South Med J. 2023;116(3):312–316. doi: 10.14423/SMJ.0000000000001525 PMC9973351 36863054

[402] Choo EK , Woods R , Walker ME , et al. The quality of assessment for learning score for evaluating written feedback in anesthesiology postgraduate medical education: a generalizability and decision study. Can Med Educ J. 2023;14(6):78–85. doi: 10.36834/cmej.75876 PMC10787859 38226296

[403] Mink RB , Schwartz A , Mahan JD , et al. Level of supervision for the entrustable professional activities common to general pediatrics and the subspecialties decreases from residency to fellowship. Acad Pediatr. 2024;24(7):1025–1030. doi: 10.1016/j.acap.2024.04.004 38631477

[404] Skulsampaopol J , Ming Y , Cusimano MD . Educating residents in spine surgery: a study of entrustable professional activities in neurosurgery and orthopedic surgery. PLoS One. 2024;19(10):e0311444. doi: 10.1371/journal.pone.0311444 PMC11451968 39365796

[405] Monti M , Pittet V , Frick S , et al. A multi-method approach to drafting candidate entrustable professional activities for a general internal Medicine residency programme. Swiss Med Wkly. 2024;154:3592. doi: 10.57187/s.3592 38801750

[406] Schwitz F , Brodmann Maeder M , Hennel EK . Competency-based education - the reform of postgraduate medical training in Switzerland. GMS J Med Educ. 2024;41(5):Doc62. 10.3205/zma001717 PMC11656186 39711866

[407] Marwaha M , Bhalla R , Rao S , et al. Minimum standard assessment of informed consent for internal Medicine transition to residency program: a cohort study. Health Sci Rep. 2023;6(9):e1523. doi: 10.1002/hsr2.1523 PMC10477888 37674619

[408] Seed JD , Gauthier S , Zevin B , et al. Simulation vs workplace-based assessment in resuscitation: a cross-specialty descriptive analysis and comparison. Can Med Educ J. 2023;14(3):92–98. doi: 10.36834/cmej.73692 PMC10351640 37465738

[409] Amare EM , Siebeck M , Yigzaw T , et al. Differences in perceptions of capability, autonomy, and expectations between residents and surgical team members in executing EPAs in Ethiopian medical education. Heliyon. 2023;9(3):e14316. doi: 10.1016/j.heliyon.2023.e14316 PMC10023974 36942250

[410] Schmidbauer ML , Pinilla S , Kunst S , et al. Fit for service: preparing residents for neurointensive care with entrustable professional activities: a delphi study. Neurocrit Care. 2024;40(2):645–653. doi: 10.1007/s12028-023-01799-x PMC10959831 37498455

[411] Baumann L , Latal B , Seiler M , et al. Paediatric rotations in undergraduate medical education in Switzerland: meeting students' expectations and the goals of the competency-based learning catalogue PROFILES. GMS J Med Educ. 2024;41(5):Doc63. 10.3205/zma001718 PMC11656168 39711865

[412] Clement EA , Oswald A , Ghosh S , et al. Exploring the quality of feedback in entrustable professional activity narratives across 24 residency training programs. J Grad Med Educ. 2024;16(1):23–29. doi: 10.4300/JGME-D-23-00210.1 PMC10829927 38304587

[413] Bryant BH , Anderson SR , Brissette M , et al. Leveraging faculty development to support validation of entrustable professional activities assessment tools in anatomic and clinical pathology training. Acad Pathol. 2024;11(2):100111. doi: 10.1016/j.acpath.2024.100111 PMC10978475 38560424

[414] Leroy PL , Krauss BS , Costa LR , et al. Procedural sedation competencies: a review and multidisciplinary international consensus statement on knowledge, skills, training, and credentialing. Br J Anaesth. 2025;134(3):817–829. doi: 10.1016/j.bja.2024.07.036 PMC11867087 39327154

[415] Cheung WJ , Bhanji F , Gofton W , et al. Design and implementation of a national program of assessment model - integrating entrustable professional activity assessments in Canadian specialist postgraduate medical education. Perspect Med Educ. 2024;13(1):44–55. doi: 10.5334/pme.956 PMC10854461 38343554

[416] Qayumi K , Masoumian Hosseini ST , Masoumian Hosseini M , et al. Enhancing medical training in conflict zones and remote areas through innovation: introducing the Canadian virtual medical university initiative. EClinicalMedicine. 2024;76:102854. doi: 10.1016/j.eclinm.2024.102854 PMC11466554 39391016

[417] Strand AC , Gingerich A , Daniels VJ . Misbehavior or misalignment? Examining the drift towards bureaucratic box-ticking in competency-based medical education. PLoS One. 2025;20(1):e0313021. doi: 10.1371/journal.pone.0313021 PMC11694975 39746080

[418] Hoffman M , Dedow L , Boscamp J . Competency-based and less time-bound: a new approach to the macro-structure of a medical school curriculum. Med Educ Online. 2024;29(1):2343205. doi: 10.1080/10872981.2024.2343205 PMC11022905 38626425

[419] Trang K , Pierce L , Wick EC , et al. Promoting resident education priorities with an acute care surgery service dashboard. J Surg Educ. 2025;82(2):103342. doi: 10.1016/j.jsurg.2024.103342 39644700

[420] Rowe KA , Ouchi K , Kennedy M , et al. Preparing preclinical medical students for routine code status discussions: a mixed-methods study. J Pain Symptom Manage. 2024;67(2):138–146. doi: 10.1016/j.jpainsymman.2023.10.017 PMC11267244 37863372

[421] Palsson P , Cederborg A , Johansson M , et al. Clinical supervisors' experience of a first-time application of entrustable professional activities in clinical supervision of medical students: findings from a Swedish pilot study. BMC Med Educ. 2024;24(1):297. doi: 10.1186/s12909-024-05211-w PMC10943870 38491437

[422] Amare EM , Siyoum MT , Abubeker FA , et al. Designing the future of medical education: the EPA framework as a catalyst to inform family planning and reproductive health fellowship training program in Ethiopia medical education: an exploratory sequential mixed method study. Adv Med Educ Pract. 2024;15:189–200. doi: 10.2147/AMEP.S438315 PMC10949272 38505496

[423] Lee Y , Khamar J , Samarasinghe Y , et al. The surgical education checklist: a novel tool to improve the use of entrustable professional activities in operative training in competence by design. J Surg Educ. 2025;82(5):103470. doi: 10.1016/j.jsurg.2025.103470 39956035

[424] Li M , Kurahashi AM , Kawaguchi S , et al. When words are your scalpel, what and how information is exchanged May be differently salient to assessors. Med Educ. 2024;58(11):1324–1332. doi: 10.1111/medu.15458 38850193

[425] Chopra S , Harley JM , Keuhl A , et al. Understanding emotions impacted by new assessment mandates implemented in medical education: a survey of residents and faculty across multiple specialties. Cureus. 2024;16(6):e62013. doi: 10.7759/cureus.62013 PMC11233152 38983997

[426] Sigurdsson V , Ten Cate O . Do summative entrustment decisions actually lead to entrustment? Clin Teach. 2024;21(1):e13668. doi: 10.1111/tct.13668 37817015

[427] Brocke TK , Eaton Jr. DB , Johnson CE , et al. Doing less later: seniorization of general surgery resident operative experience in the entrustable professional activities at veterans affairs hospitals. J Surg Educ. 2025;82(3):103403. doi: 10.1016/j.jsurg.2024.103403 39799710

[428] Cox ML , Weaver ML , Johnson C , et al. Early findings and strategies for successful implementation of SIMPL workplace-based assessments within vascular surgery residency and fellowship programs. J Vasc Surg. 2023;78(3):806–14 e2. doi: 10.1016/j.jvs.2023.04.039 37164236

[429] Johnson D , Chopra S , Bilgic E . Exploring the use of natural language processing to understand emotions of trainees and faculty regarding entrustable professional activity assessments. J Grad Med Educ. 2024;16(3):323–327. doi: 10.4300/JGME-D-23-00526.1 PMC11173031 38882409

[430] Kinston R , Gay S , McKinley RK , et al. How well do UK assistantships equip medical students for graduate practice? Think EPAs. Adv Health Sci Educ Theory Pract. 2024;29(1):173–198. doi: 10.1007/s10459-023-10249-4 PMC10927851 37347459

[431] Thoonen BPA , Scherpbier-de Haan ND , Fluit C , et al. How do trainees use EPAs to regulate their learning in the clinical environment? A grounded theory study. Perspect Med Educ. 2024;13(1):431–441. doi: 10.5334/pme.1403 PMC11378707 39247555

[432] Lin YH , Yang YY , Chen CH , et al. Can routine EPA-based assessments predict OSCE performances of undergraduate medical students? Med Teach. 2024;1–10. 10.1080/0142159X.2024.2413024 39400116

[433] Ryan MS , Gielissen KA , Shin D , et al. How well do workplace-based assessments support summative entrustment decisions? A multi-institutional generalisability study. Med Educ. 2024;58(7):825–837. doi: 10.1111/medu.15291 38167833

[434] Peters H , Garbe A , Breil SM , et al. Big five personality traits of medical students and workplace performance in the final clerkship year using an EPA framework. BMC Med Educ. 2024;24(1):453. doi: 10.1186/s12909-024-05434-x PMC11044476 38664702

[435] Paternotte E , Dijksterhuis M , Goverde A , et al. Comparison of OBGYN postgraduate curricula and assessment methods between Canada and the Netherlands: an auto-ethnographic study. Front Med (Lausanne). 2024;11:1363222. doi: 10.3389/fmed.2024.1363222 PMC11004340 38601119

[436] Chen C , Coppola KM , Weber P , et al. Interactive patient safety and quality improvement capstone during transition-to-residency program: virtual and in-person focused workshop for EPA 13. BMC Med Educ. 2023;23(1):235. doi: 10.1186/s12909-023-04220-5 PMC10091311 37046253

[437] Rowat J , Mhatre G , Suneja M . Mobile application to improve just-in-time 2-Way formative feedback in graduate medical education. J Grad Med Educ. 2024;16(2):221–226. doi: 10.4300/JGME-D-23-00378.1 PMC11234316 38993301

[438] Peters H , Holzhausen Y , Czeskleba A , et al. Defining a set of teaching EPAs tailored to an undergraduate medical curriculum using a modified delphi approach. BMC Med Educ. 2024;24(1):588. doi: 10.1186/s12909-024-05553-5 PMC11134953 38807126

[439] O'Neill R , Adeli C , San Miguel CE . An appy that needs epi: an atypical presentation of anaphylaxis. J Educ Teach Emerg Med. 2024;9(1):S1–S41. 10.21980/J80H14 PMC10854882 38344051

[440] Song J , Wei G , Cheng H , et al. Establishing a set of qualities for clinical teaching faculty in undergraduate medical internship. BMC Med Educ. 2025;25(1):298. doi: 10.1186/s12909-025-06868-7 PMC11854011 39994785

[441] Garber AM , Meliagros P , Diener-Brazelle J , et al. Using virtual reality to teach medical students cross-coverage skills. Am J Med. 2024;137(5):454–458. doi: 10.1016/j.amjmed.2024.02.002 38364987

[442] Leclair R , Ho JSS , Braund H , et al. Exploring the quality of narrative feedback provided to residents during ambulatory patient care in Medicine and surgery. J Med Educ Curric Dev. 2023;10:23821205231175734. doi: 10.1177/23821205231175734 PMC10192660 37216002

[443] Spadafore M , Yilmaz Y , Rally V , et al. Using natural language processing to evaluate the quality of supervisor narrative comments in competency-based medical education. Acad Med. 2024;99(5):534–540. doi: 10.1097/ACM.0000000000005634 38232079

[444] Ott M , Apramian T , Cristancho S , et al. Unintended consequences of technology in competency-based education: a qualitative study of lessons learned in an OtoHNS program. J Otolaryngol Head Neck Surg. 2023;52(1):55. doi: 10.1186/s40463-023-00649-2 PMC10463791 37612760

[445] Gilchrist PT , Beaton NSM , Atkin JN , et al. The new diploma of rural generalist anaesthesia: supporting Australian rural and remote communities. Anaesth Intensive Care. 2024;52(1):6–15. doi: 10.1177/0310057X231196909 38006613

[446] Alexander M , Behrend R , Franz A , et al. Feeling the responsibility: exploring the emotional experiences of final-year medical students when carrying out clinical tasks. Med Teach. 2025;47(3):513–520. doi: 10.1080/0142159X.2024.2351137 38771960

[447] Jenkins L , Mash R , Naidoo M , et al. Developing an electronic portfolio of learning for family Medicine training in South Africa. Afr J Prim Health Care Fam Med. 2024;16(1):e1–e4. doi: 10.4102/PHCFM.v16i1.4525 PMC11219992 38949444

[448] Tessmann T , Marty AP , Stricker D , et al. There is no "too small" for frequent workplace-based assessment: differences between large and small residency programs in anesthesia when using a mobile application to assess EPAs. GMS J Med Educ. 2024;41(5):Doc54 10.3205/zma001709 PMC11656171 39711857

[449] Curtis R , Moon CC , Hanmore T , et al. Prompting better feedback: investigating the effect of targeted form design on quality of narrative feedback in ophthalmology CBME assessments. Can J Ophthalmol. 2024 10.1016/j.jcjo.2024.10.014 39528224

[450] Nguyen-Tri I , Tremblay-Laroche D , Lavigne F , et al. Feedback in an entrustment-based objective structured clinical examination: analysis of content and scoring methods. J Grad Med Educ. 2024;16(3):286–295. doi: 10.4300/JGME-D-23-00569.1 PMC11173042 38882423

[451] Rainer R , Reynolds BC , Adeli C , et al. Subarachnoid hemorrhage causing a seizure: an assessment simulation for medical students. J Educ Teach Emerg Med. 2024;9(3):S30–S64. 10.21980/J8XH1H PMC11312874 39129724

[452] Millar JK , Matusko N , Evans J , et al. Faculty entrustment and resident entrustability. JAMA Surg. 2024;159(3):277–285. doi: 10.1001/jamasurg.2023.6915 PMC10782383 38198146

[453] Tanaka P , Marty A , Park YS , et al. Defining entrustable professional activities for first year anesthesiology residents: a delphi study. J Clin Anesth. 2023;88:111116. doi: 10.1016/j.jclinane.2023.111116 37278050

[454] Rajaragupathy S , Govindarajan S , Ganesan J , et al. Identifying entrustable professional activities for MD program in biochemistry-A modified delphi approach. Biochem Mol Biol Educ. 2023;51(4):402–409. doi: 10.1002/bmb.21736 37148494

[455] Langhan ML , Boyer DL , Hsu D , et al. Implementing entrustable professional activities in pediatric fellowships: facilitating the process. Pediatrics. 2024;153(6). doi: 10.1542/peds.2023-065024 38757175

[456] Brasel KJ , Lindeman B , Jones A , et al. Implementation of entrustable professional activities in general surgery: results of a national pilot study. Ann Surg. 2023;278(4):578–586. doi: 10.1097/SLA.0000000000005991 37436883

[457] Kawaguchi S , Myers J , Li M , et al. Entrustable professional activities in palliative Medicine: a faculty and learner development activity. J Palliat Med. 2024;27(8):1055–1059. doi: 10.1089/jpm.2023.0682 39007218

[458] McClintock AH , Rusiecki J , Casas RS , et al. Special report: entrustable professional activities in Sex- and gender-based Women's health. J Gen Intern Med. 2025;40(1):82–88. doi: 10.1007/s11606-024-08843-2 PMC11780014 38858342

[459] Dent DL , Patnaik R , Atkinson A , et al. Implementation of entrustable professional activities in multiple surgical residencies: a quality improvement approach. Am J Surg. 2023;226(6):868–872. doi: 10.1016/j.amjsurg.2023.07.014 37507253

[460] McDonald J , Tobin S , Parsons C , et al. Entrustable professional activities and feedback for learning: insights from a mixed methods study. Clin Teach. 2025;22(1):e13837. doi: 10.1111/tct.13837 39505496

[461] Goldowsky A , Billings W , Kickel A , et al. @MondayNightIBD and the expanding gastroenterology twitterverse: a study on continuing medical education on #GITwitter. Am J Gastroenterol. 2023;118(5):855–860. 10.14309/ajg.0000000000002103 36623175

[462] Williams-Karnesky RL , White E , Holmstrom A , et al. Faculty and resident perspectives on the implementation of entrustable professional activities in general surgery residency. J Surg Educ. 2024;81(6):841–849. doi: 10.1016/j.jsurg.2024.03.006 38664173

[463] Zmijewski P , McLeod MC , Akhund R , et al. Entrustable professional activities in endocrine surgery: a national pilot study. Surgery. 2025;177:108860. doi: 10.1016/j.surg.2024.09.018 39505596

[464] Montgomery KB , Mellinger JD , McLeod MC , et al. Decision-making confidence of clinical competency committees for entrustable professional activities. JAMA Surg. 2024;159(7):801–808. doi: 10.1001/jamasurg.2024.0809 PMC11079788 38717759

[465] Lee MC , Melcer EF , Merrell SB , et al. Usability of ENTRUST as an assessment tool for entrustable professional activities (EPAs): a mixed methods analysis. J Surg Educ. 2023;80(11):1693–1702. doi: 10.1016/j.jsurg.2023.09.001 37821350

[466] Eliasz KL , Nick MW , Zabar S , et al. Viewing readiness-for-residency through binoculars: mapping competency-based assessments to the AAMC's 13 core entrustable professional activities (EPAs). Teach Learn Med. 2023;35(4):436–441. doi: 10.1080/10401334.2022.2082432 35668557

[467] Stumbar SE , Eliason S , Toonkel R , et al. Next steps for workplace-based assessments of entrustable professional activities. Clin Teach. 2024;21(4):e13739. doi: 10.1111/tct.13739 38311985

[468] Hanson-Kahn A , Rowe-Teeter C , Siskind C , et al. Proposed use of entrustable professional activities (EPAs) in genetic counseling for clinical training and assessment. J Genet Couns. 2024;33(1):164–167. doi: 10.1002/jgc4.1871 38356180

[469] Jones-Carr ME , McLeod C , Baker S , et al. Framing our expectations: variability in entrustable professional activity assessments. J Surg Educ. 2024;81(10):1355–1361. doi: 10.1016/j.jsurg.2024.07.025 39163720

[470] Langhan ML , Stafford DEJ , Myers AL , et al. Clinical competency committee perceptions of entrustable professional activities and their value in assessing fellows: a qualitative study of pediatric subspecialty program directors. Med Teach. 2023;45(6):650–657. doi: 10.1080/0142159X.2022.2147054 36420760

[471] van Keulen SG , de Raad T , Raymakers-Janssen P , et al. Exploring interprofessional development of entrustable professional activities for pediatric intensive care fellows: a proof-of-concept study. Teach Learn Med. 2024;36(2):154–162. doi: 10.1080/10401334.2023.2200760 37071751

[472] Abhijita B , Sinha A , Choudhury AA , et al. Effectiveness of entrustable professional activity-based clinical psychiatry training for Indian medical interns: a preliminary study. Psychiatry. 2024;87(1):96–98. doi: 10.1080/00332747.2023.2291959 38227497

[473] Safavi AH , Sienna J , Strang BK , et al. Competency-based medical education in Canadian radiation oncology residency training: an institutional implementation pilot study. J Cancer Educ. 2023;38(1):274–284. doi: 10.1007/s13187-021-02112-0 34859361

[474] Jeans EB , Brower JV , Burmeister JW , et al. Development of a United States radiation oncology curricular framework: a stakeholder delphi consensus. Int J Radiat Oncol Biol Phys. 2023;115(5):1030–1040. doi: 10.1016/j.ijrobp.2022.12.009 36549345

[475] Imm MR , Agarwal G , Zhang C , et al. EPMO: a novel medical student assessment tool that integrates entrustable professional activities, prime, and the modified Ottawa coactivity scale. Med Teach. 2023;45(4):419–425. doi: 10.1080/0142159X.2022.2137012 36288734

[476] Gielissen KA , Foust A , Weinstein AR , et al. How internal Medicine clerkship directors are using entrustable professional activities: a national survey study. J Gen Intern Med. 2025;40(1):46–53. doi: 10.1007/s11606-024-08991-5 PMC11780069 39147939

[477] Chin M , Pack R , Cristancho S . A whole other competence story": exploring faculty perspectives on the process of workplace-based assessment of entrustable professional activities. Adv Health Sci Educ Theory Pract. 2023;28(2):369–385. doi: 10.1007/s10459-022-10156-0 35997910

[478] Bresler T , Stiles E , Schaeztel S , et al. Oral examination of fourth-year medical students on surgery rotations allows faculty to assess core entrustable professional activities for entering residency (CEPAR): proof-of-concept and analysis of student submissions. Am J Surg. 2023;225(5):841–846. doi: 10.1016/j.amjsurg.2023.01.020 36764899

[479] Hsu DC , Baghdassarian A , Caglar D , et al. Pediatric emergency Medicine fellowship program Directors' viewpoint: minimum levels of entrustment for graduating fellows and practicing physicians to perform the Subspecialty's professional activities. Pediatr Emerg Care. 2023;39(8):574–579. doi: 10.1097/PEC.0000000000002817 35947053

[480] Fernandes RD , de Vries I , McEwen L , et al. Evaluating the quality of narrative feedback for entrustable professional activities in a surgery residency program. Ann Surg. 2024;280(6):916–924. doi: 10.1097/SLA.0000000000006308 38660808

[481] Phares A , Binda C , Joharifard S , et al. Establishing a competency based medical education curriculum for international medical graduates pursuing pediatric surgery training in high-income countries. J Pediatr Surg. 2025;60(4):162227. doi: 10.1016/j.jpedsurg.2025.162227 39954319

[482] Colbert-Getz JM , Lappe K , Gerstenberger J , et al. Capturing growth curves of medical students' clinical skills performance. Clin Teach. 2023;20(6):e13623. doi: 10.1111/tct.13623 37605795

[483] Wong L , Chung AD , Rogoza C , et al. Peering into the future: a first look at the CBME transition to practice stage in diagnostic radiology. Acad Radiol. 2023;30(10):2406–2417. doi: 10.1016/j.acra.2023.06.013 37453881

[484] Costello LL , Cho DD , Daniel RC , et al. Emergency Medicine resident perceptions of simulation-based training and assessment in competence by design. CJEM. 2023;25(10):828–835. doi: 10.1007/s43678-023-00577-0 37665550

[485] Chang C , Jesneck J , Lee E , et al. EHR-Driven delivery of EPA assessments significantly improves participation. J Surg Educ. 2024;81(11):A12–A22. doi: 10.1016/j.jsurg.2024.06.015 38964960

[486] Harvey A , Paget M , McLaughlin K , et al. How much is enough? Proposing achievement thresholds for core EPAs of graduating medical students in Canada. Med Teach. 2023;45(9):1054–1060. doi: 10.1080/0142159X.2023.2215910 37262177

[487] Brown A , La J , Keri MI , et al. In EPAs we trust, is quality and safety a must? A cross-specialty analysis of entrustable professional activity guides. Med Teach. 2025;47(1):134–142. doi: 10.1080/0142159X.2024.2323177 38527417

[488] Woodworth GE , Goldstein ZT , Ambardekar AP , et al. Development and pilot testing of a programmatic system for competency assessment in US anesthesiology residency training. Anesth Analg. 2024;138(5):1081–1093. doi: 10.1213/ANE.0000000000006667 37801598

[489] Jensen RM , Kearse LE , Anand A , et al. The program director perspective: perceptions of PGY5 Residents' operative self-efficacy and entrustment. Am Surg. 2023;89(7):3098–3103. doi: 10.1177/00031348231157862 36802912

[490] White EM , Esposito AC , Yoo PS . Should obtaining informed consent be considered an entrustable professional activity? Insights from whether and how attendings entrust surgical trainees. Acad Med. 2024;99(8):897–903. doi: 10.1097/ACM.0000000000005587 38113443

[491] Nierenberg DW , Lewis LD . The clinical pharmacology and therapeutics course for graduating medical students at dartmouth: preparation for internship and residency. Br J Clin Pharmacol. 2024;91:2162–2167. doi: 10.1111/bcp.16179 39077813

[492] Plash WB , Leiva DF , Watkins KD , et al. Curriculum guidelines for wilderness Medicine medical student electives: 2025 update. Wilderness Environ Med. 2025;10806032251318582. doi: 10.1177/10806032251318582 39982027

[493] Miller F , Wood S , Livingston P . The lived experience of competence by design: Canadian resident physicians' perspectives. Can J Anaesth. 2024;71(2):254–263. doi: 10.1007/s12630-023-02678-x 38133715

[494] Chlebowski MM , Migally K , Werho DK , et al. Cardiac critical care fellowship training in the United States and Canada: pediatric cardiac intensive care society-endorsed subcompetencies to the 2022 entrustable professional activities. Pediatr Crit Care Med. 2024;25(6):e303–e9. doi: 10.1097/PCC.0000000000003464 38329380

[495] Shuford A , Carney PA , Ketterer B , et al. An analysis of workplace-based assessments for core entrustable professional activities for entering residency: does type of clinical assessor influence level of supervision ratings? Acad Med. 2024;99(8):904–911. doi: 10.1097/ACM.0000000000005691 38498305

[496] Caretta-Weyer HA , Park YS , Tekian A , et al. Identifying emergency Medicine program directors' expectations of competence upon entry into residency: bridging the distance from the association of American medical colleges core entrustable professional activities. AEM Educ Train. 2025;9(2):e70024. doi: 10.1002/aet2.70024 PMC11897532 40083335

[497] Layne D , Jung S , Varley P , et al. How well do faculty do in providing general surgery EPA feedback? Am J Surg. 2024;236:115902. doi: 10.1016/j.amjsurg.2024.115902 39242235

[498] Kaur B , Taylor EM . Development of a pediatric anesthesia fellowship curriculum in australasia by the society for pediatric anesthesia of New Zealand and Australia (SPANZA) education sub committee. Paediatr Anaesth. 2023;33(2):100–106. doi: 10.1111/pan.14536 35876724

[499] Schumacher DJ , Winn A , Hemond J , et al. Associations between residency program size and readiness for unsupervised practice in pediatrics. Acad Pediatr. 2025;25:102806. doi: 10.1016/j.acap.2025.102806 40064464

[500] Donohue K , Davis N , Farber D , et al. An internal Medicine residency preparatory curriculum for fourth-year medical students. Clin Teach. 2024;21(2):e13703. doi: 10.1111/tct.13703 38049309

[501] Go MR , Traugott AL , Ejaz A , et al. Measuring chief resident skill and entrustment progression in an operative coaching program: four Years' experience. J Surg Educ. 2024;81(4):457–464. doi: 10.1016/j.jsurg.2023.12.014 38388313

[502] Braund H , Dagnone JD , Hall AK , et al. Competency based medical education implementation at the institutional level: a cross-discipline comparative program evaluation. Med Teach. 2025;47(4):705–712. doi: 10.1080/0142159X.2024.2362909 39101833

[503] Karnik R , Robinson B , Glickstein J , et al. Minimum supervision levels required for pediatric cardiology fellowship graduation by pediatric cardiology fellowship program directors. Pediatr Cardiol. 2025;46(1):181–188. doi: 10.1007/s00246-023-03371-y 38117291

[504] Pimentel J , Garcia JC , Romero-Tapia AE , et al. Competency-based cultural safety training in medical education at la sabana university, Colombia: a roadmap of curricular modernization. Teach Learn Med. 2025;37(1):127–136. doi: 10.1080/10401334.2023.2246964 37929697

[505] Jones ML , Silk H , Savageau JA . A case study on development of a spiral oral health curriculum for medical students. J Dent Educ. 2024;89:853–861. doi: 10.1002/jdd.13769 39533534

[506] Huber L , Good R , Bone MF , et al. A modified delphi study for curricular content of simulation-based medical education for pediatric residency programs. Acad Pediatr. 2024;24(5):856–865. doi: 10.1016/j.acap.2024.04.008 38663801

[507] Wong LY , Lam N , Son YA , et al. Correlation of performance on the ENTRUST assessment platform with other variables in competency-based surgical education. J Surg Educ. 2025;82(1):103293. doi: 10.1016/j.jsurg.2024.09.012 39532037

[508] Tanaka P , Soo Park Y , Chen CY , et al. Domains influencing faculty decisions on the level of supervision required for anesthesiology EPAs with analysis of feedback comments. J Surg Educ. 2024;81(5):741–752. doi: 10.1016/j.jsurg.2024.02.003 38553368

[509] Skulsampaopol J , Ming Y , Cusimano MD . Establishing competency assessment standards for graduating neurosurgery, plastic surgery, and orthopedic surgery residents in peripheral nerve surgery. Oper Neurosurg (Hagerstown). 2025;29:686–695. doi: 10.1227/ons.0000000000001521 40042278

[510] Ginsburg S , Stroud L , Brydges R , et al. Dual purposes by design: exploring alignment between residents' and academic advisors' documents in a longitudinal program. Adv Health Sci Educ Theory Pract. 2024;29(5):1631–1647. doi: 10.1007/s10459-024-10318-2 38438699

[511] Adrales G , Ardito F , Chowbey P , et al. Laparoscopic cholecystectomy critical view of safety (LC-CVS): a multi-national validation study of an objective, procedure-specific assessment using video-based assessment (VBA). Surg Endosc. 2024;38(2):922–930. doi: 10.1007/s00464-023-10479-y 37891369

[512] McEllistrem B , Hennus MP , Fawns T , et al. Exploring the Irish general practice training community's perceptions on how an entrustable professional activities dashboard implementation could facilitate general practice training in Ireland. Educ Prim Care. 2023;34(2):91–99. doi: 10.1080/14739879.2023.2191340 36960834

[513] Ravishankar M , Areti A , Kumar VRH , et al. Competency-based postgraduate training: mentoring and monitoring using entrustable professional activities with descriptive rubrics for objectivity- A step beyond dreyfus. Natl Med J India. 2023;36(3):176–181. 10.25259/NMJI_860_2021 38692622

[514] Sahi N , Humphrey-Murto S , Brennan EE , et al. Current use of simulation for EPA assessment in emergency Medicine. CJEM. 2024;26(3):179–187. doi: 10.1007/s43678-024-00649-9 38374281

[515] Sathe TS , Shah M , Pokrzywa CJ , et al. An open operative readiness standard for surgical trainees. J Surg Res. 2024;293:281–290. doi: 10.1016/j.jss.2023.09.017 37804798

[516] Suneja M , Hanrahan KD , Kreiter C , et al. Psychometric properties of entrustable professional activity-based objective structured clinical examinations during transition from undergraduate to graduate medical education: a generalizability study. Acad Med. 2025;100(2):179–183. doi: 10.1097/ACM.0000000000005719 38534117

[517] Millham LRI , Potter J , Hirsh DA , et al. Incorporation of trauma-informed care into entrustable professional activities for medical student assessment. Acad Med. 2025;100(3):290–294. doi: 10.1097/ACM.0000000000005824 39042418

[518] Lin Y , Cheng A , Hecker K , et al. Implementing economic evaluation in simulation-based medical education: challenges and opportunities. Med Educ. 2018;52(2):150–160. doi: 10.1111/medu.13411 28949032

[519] Jones RF , Korn D . On the cost of educating a medical student. Acad Med. 1997;72(3):200–210. doi: 10.1097/00001888-199703000-00015 9075424

[520] Godwin M , Ruhland L , Casson I , et al. Pragmatic controlled clinical trials in primary care: the struggle between external and internal validity. BMC Med Res Methodol. 2003;3:28. doi: 10.1186/1471-2288-3-28 PMC317298 14690550

[521] Harrison R , Jones B , Gardner P , et al. Quality assessment with diverse studies (QuADS): an appraisal tool for methodological and reporting quality in systematic reviews of mixed- or multi-method studies. BMC Health Serv Res. 2021;21(1):144. doi: 10.1186/s12913-021-06122-y PMC7885606 33588842

[522] Cervero RM , Gaines JK . The impact of CME on physician performance and patient health outcomes: an updated synthesis of systematic reviews. J Contin Educ Health Prof. 2015;35(2):131–138. doi: 10.1002/chp.21290 26115113

[523] Baldwin CD , Chandran L , Gusic ME . Building sustainable professional development programs: applying strategies from implementation science to translate evidence into practice. J Contin Educ Health Prof. 2017;37(2):106–115. doi: 10.1097/CEH.0000000000000151 28562499

[524] Lupi CS , Ownby AR , Jokela JA , et al. Faculty development revisited: a systems-based view of stakeholder development to meet the demands of entrustable professional activity implementation. Acad Med. 2018;93(10):1472–1479. doi: 10.1097/ACM.0000000000002297 29794524

[525] Marty AP , Linsenmeyer M , George B , et al. Mobile technologies to support workplace-based assessment for entrustment decisions: guidelines for programs and educators: AMEE guide no. 154. Med Teach. 2023;45(11):1203–1213. doi: 10.1080/0142159X.2023.2168527 36706225

[526] Cook DA , West CP . Perspective: reconsidering the focus on "outcomes research" in medical education: a cautionary note. Acad Med. 2013;88(2):162–167. doi: 10.1097/ACM.0b013e31827c3d78 23269304

[527] O'Malley PG , Pangaro LN . Research in medical education and patient-centered outcomes: shall ever the twain meet? JAMA Intern Med. 2016;176(2):167–168. doi: 10.1001/jamainternmed.2015.6938 26641544

