# Supplementary material for: Entrustable professional activities in medical education: strategies and results - a systematic review
Source: Med Educ Online. 2026 Oct 10;31(1):2740871. doi: 10.1080/10872981.2026.2740871 (PMC13656657; doi:10.1080/10872981.2026.2740871)
Supplement: Supplementary file 1 [file ZMEO_A_2740871_SM1038.docx]

| Table 2. Journal of publication and Quartile analysis | | | |
| --- | --- | --- | --- |
|  | **Number of studies** | **% of Studies** | **Quartile** |
| Name of Journal of Publication | | | |
| Acad Med | 48 | 10,0% | 1 |
| BMC Med Educ | 31 | 6,5% | 1 |
| J Surg Educ | 26 | 5,4% | 1 |
| J Grad Med Educ | 24 | 5,0% | 2 |
| Med Teach | 17 | 3,6% | 1 |
| GMS J Med Educ | 13 | 2,7% | 2 |
| Acad Pediatr | 13 | 2,7% | 1 |
| Perspect Med Educ | 12 | 2,5% | 1 |
| J Gen Intern Med | 12 | 2,5% | 2 |
| Clin Teach | 10 | 2,1% | 2 |
| Acad Pathol | 8 | 1,7% | 3 |
| Am J Surg | 8 | 1,7% | 1 |
| Can Med Educ J | 8 | 1,7% | 3 |
| AEM Educ Train | 7 | 1,5% | 2 |
| Cureus | 7 | 1,5% | 3 |
| Med Educ | 7 | 1,5% | 1 |
| Med Educ Online | 7 | 1,5% | 1 |
| Teach Learn Med | 7 | 1,5% | 1 |
| CJEM | 6 | 1,3% | 2 |
| J Med Educ Curric Dev | 6 | 1,3% | 4 |
| Med Sci Educ | 6 | 1,3% | 3 |
| Journals of Publication of <1% of the studies* | 195 | 40,8% |  |
| TOTAL | **478** |  |  |
|  | | | |
| Quartile Analysis of the Scientific Journals used for publication | | | |
| Quartile | **Number of Journals** | **% of Journals** | |
| Q1 | 74 | 48,0% | |
| Q2 | 48 | 31,2% | |
| Q3 | 22 | 14,3% | |
| Q4 | 5 | 3,2% | |
| No Information (NI) | 5 | 3,2% | |
| Total | 154 | 100% | |

* Journals of Publication of <1% of the studies**:** Acad Forensic Pathol, Acad Psychiatry, Acad Radiol, Acta Med Indones, Acta Neurochir (Wien), Adv Health Sci Educ Theory Pract, Adv Med Educ Pract, Adv Simul (Lond), Afr J Prim Health Care Fam Med, Am J Gastroenterol, Am J Kidney Dis, Am J Med, Am J Phys Med Rehabil, Am Surg, Anaesth Intensive Care, Anesth Analg, Ann Intern Med, Ann Surg, ANZ J Surg, Arch Argent Pediatr, Arch Esp Urol, Arch Pathol Lab Med, Arthritis Care Res (Hoboken), ATS Sch, Australas Psychiatry, Biochem Mol Biol Educ, BMC Anesthesiol, BMJ Glob Health, BMJ Open, BMJ Simul Technol Enhanc Learn, Br J Anaesth, Br J, Clin Pharmacol, Can Assoc Radiol J, Can Geriatr J, Can J Anaesth, Can J Kidney Health Dis, Can J Neurol Sci, Can J Ophthalmol, Can Urol Assoc J, Chest, Cir Cir, Clin Orthop Relat Res, Curr Probl, Diagn Radiol, Dig Dis Sci, EClinicalMedicine, Educ Prim Care, Eur J Anaesthesiol, Eur J Surg Oncol, Eur Urol Open Sci, Fam Med, Front Med (Lausanne), Front Pediatr, Front Public Health, Future Healthc J, Games Health J, Gastroenterology, Gerontol Geriatr Educ, Health Sci Rep, Heliyon, Hosp Pediatr, HRB Open Res, Ind Psychiatry J, Indian J Otolaryngol Head Neck Surg, Indian J Public Health, Inflamm Bowel Dis, Insights Imaging, Int J Adolesc Med Health, Int J, Geriatr Psychiatry, Int J Radiat Oncol Biol Phys, Ir J Med Sci, J Acute Med, J Am Coll Surg, J Am Geriatr Soc, J Am Osteopath Assoc, J Cancer Educ, J Clin Anesth, J CME, J Crit Care, J Cytol, J Dent Educ, J Educ Eval Health Prof, J Educ Teach Emerg Med, J Genet Couns, J Lab Physicians

J Med Internet Res, J Nepal Health Res Counc, J Neurooncol, J Obstet Gynaecol Can, J Otolaryngol Head Neck Surg, J Pain Symptom Manage, J Palliat Med, J Pediatr (Rio J), J Pediatr Gastroenterol Nutr, J Pediatr Surg, J Perinatol, J Spinal Cord Med, J Surg Res, J Vasc Surg, JAMA Netw Open, JAMA Surg, JAMMI, JMIR Med Educ, Journal of Surgical Education, Med J Armed Forces India, Med J Aust, MedEdPORTAL, Medicina (B Aires), Natl Med J India, Neurocrit Care

Oper Neurosurg (Hagerstown), Otolaryngol Head Neck Surg, Paediatr Anaesth, Pediatr Cardiol, Pediatr Clin North Am, Pediatr Crit Care Med, Pediatr Emerg Care, Pediatrics, PeerJ, PLoS One, PRiMER, Psychiatry, R I Med J (2013), Radiother Oncol, Rehabil Res Pract, South Med J, Surg Endosc, Surg Open Sci, Surgery, Swiss Med Wkly, Transfusion, Virchows Arch, West J Emerg Med, Wilderness Environ Med
