# Supplementary material for: Entrustable professional activities in medical education: strategies and results - a systematic review
Source: Med Educ Online. 2026 Oct 10;31(1):2740871. doi: 10.1080/10872981.2026.2740871 (PMC13656657; doi:10.1080/10872981.2026.2740871)
Supplement: Supplementary file 2 [file ZMEO_A_2740871_SM1039.docx]

| Table 13. Quality assessment using QATSDD | | | | | |
| --- | --- | --- | --- | --- | --- |
|  |  |  |  |  |  |
|  | **Number of studies** | **% of Studies** |  |  |  |
| QATSDD Type of study | | | | | |
| Qualitative | 227 | 47.5% |  |  |  |
| Quantitative | 171 | 35.8% |  |  |  |
| Mixed | 80 | 16.7% |  |  |  |
|  |  |  |  |  |  |
|  |  |  |  |  |  |
| % of maximum possible QATSDD score statistics | | | | | |
| N | Valid | 478 |  |  |  |
|  | Missing | 0 |  |  |  |
| Mean | | 52.6% |  |  |  |
| Standard of Deviation (SD) | | 20.5% |  |  |  |
| Minimum | | 7.14% |  |  |  |
| Maximum | | 95.2% |  |  |  |
|  |  |  |  |  |  |
|  |  |  |  |  |  |
| QATSDD Item Analysis | | | | | |
|  | **N** | | **Mean** | **Median** | **Mode** |
|  | **Valid** | Missing |  |  |  |
| 1. Explicit theoretical framework | 478 | 0 | 1.95 | 2 | 2 |
| 2. Statement of aims/objectives in main body of report | 478 | 0 | 2.35 | 2 | 3 |
| 3. Clear description of research setting | 478 | 0 | 2.15 | 2 | 2 |
| 4. Evidence of sample size considered in terms of analysis | 478 | 0 | 0.76 | 1 | 0 |
| 5. Representative sample of target group of a reasonable size | 478 | 0 | 1.63 | 2 | 2 |
| 6. Description of procedure for data collection | 478 | 0 | 1.91 | 2 | 3 |
| 7. Rationale for choice of data collection tool(s) | 478 | 0 | 1.55 | 1.5 | 1 |
| 8. Detailed recruitment data | 478 | 0 | 0.84 | 1 | 0 |
| 9. Statistical assessment reliability and validity of measurement tool(s) (Quantitative studies only) | 251 | 227 | 1.5 | 2 | 0 |
| 10. Fit between research question and method of data collection (Quantitative studies only) | 251 | 227 | 2.57 | 3 | 3 |
| 11. Fit between research question and format and content of data collection tool (Qualitative studies only) | 307 | 171 | 1.89 | 2 | 2 |
| 12. Fit between research question and method of analysis | 478 | 0 | 2.19 | 3 | 3 |
| 13. Good justification for analytic method selected | 478 | 0 | 1.49 | 1 | 3 |
| 14. Assessment of reliability of analytic process (Qualitative studies only) | 307 | 171 | 0.92 | 0 | 0 |
| 15. Evidence of user involvement in design | 478 | 0 | 0.45 | 0 | 0 |
| 16. Strengths and limitations critically discussed | 478 | 0 | 1.47 | 2 | 2 |
|  |  |  |  |  |  |
| % of maximum possible QATSDD score and Year of Publication Correlation | | | | | |
|  | | **% of maximum possible QATSDD score** | **Year of Publication** |  |  |
| % of maximum possible QATSDD Score | Pearson Correlation | 1 | 0.534^**^ |  |  |
|  | Sig. (2 tailed) |  | <0.001 |  |  |
|  | N | 478 | 478 |  |  |
| Year of Publication | Pearson Correlation | 0.534^**^ | 1 |  |  |
|  | Sig. (2 tailed) | <0.001 |  |  |  |
|  | N | 478 | 478 |  |  |
| **. Correlation is significant at the 0.01 level (2 tailed). | | | |  |  |
