# Supplementary material for: Entrustable professional activities in medical education: strategies and results - a systematic review
Source: Med Educ Online. 2026 Oct 10;31(1):2740871. doi: 10.1080/10872981.2026.2740871 (PMC13656657; doi:10.1080/10872981.2026.2740871)
Supplement: Supplementary file 3 [file ZMEO_A_2740871_SM1040.docx]

Entrustable Professional Activities (EPAs) in medical education: strategies and results. A systematic review

Search strategy:

Due to the specific nature of the scope of interest of this project, search strategy will be based on the terms “entrustable professional activity” and “entrustable professional activities” and “EPAs” linked with the Boolean operator “OR” and on the term “Medical education” linked with the Boolean operator “AND”.

Example of search strategy proposed for PubMed database:

((EPAs[Title/Abstract]) OR (entrustable professional activities[Title/Abstract]) OR (entrustable professional activity[Title/Abstract]) AND (english[Filter] OR portuguese[Filter] OR spanish[Filter] OR French [Filter])) AND (("Education, Medical"[Mesh]) OR (medical education) AND (english[Filter] OR portuguese[Filter] OR spanish[Filter] OR French [Filter]))

The keywords considered will be: ENTRUSTABLE PROFESSIONAL ACTIVITIES, MEDICAL EDUCATION
